# KLF4 is a therapeutically tractable brake on fibroblast activation that promotes resolution of pulmonary fibrosis

**DOI:** 10.1172/jci.insight.160688

**Published:** 2022-08-22

**Authors:** Loka R. Penke, Jennifer M. Speth, Steven K. Huang, Sean M. Fortier, Jared Baas, Marc Peters-Golden

**Affiliations:** Division of Pulmonary and Critical Care Medicine, Department of Internal Medicine, University of Michigan Medical School, Ann Arbor, Michigan, USA.

**Keywords:** Pulmonology, Fibrosis

## Abstract

There is a paucity of information about potential molecular brakes on the activation of fibroblasts that drive tissue fibrosis. The transcription factor Krüppel-like factor 4 (KLF4) is best known as a determinant of cell stemness and a tumor suppressor. We found that its expression was diminished in fibroblasts from fibrotic lung. Gain- and loss-of-function studies showed that KLF4 inhibited fibroblast proliferation, collagen synthesis, and differentiation to myofibroblasts, while restoring their sensitivity to apoptosis. Conditional deletion of KLF4 from fibroblasts potentiated the peak degree of pulmonary fibrosis and abrogated the subsequent spontaneous resolution in a model of transient fibrosis. A small molecule inducer of KLF4 was able to restore its expression in fibrotic fibroblasts and elicit resolution in an experimental model characterized by more clinically relevant persistent pulmonary fibrosis. These data identify KLF4 as a pivotal brake on fibroblast activation whose induction represents a therapeutic approach in fibrosis of the lung and perhaps other organs.

## Graphical abstract

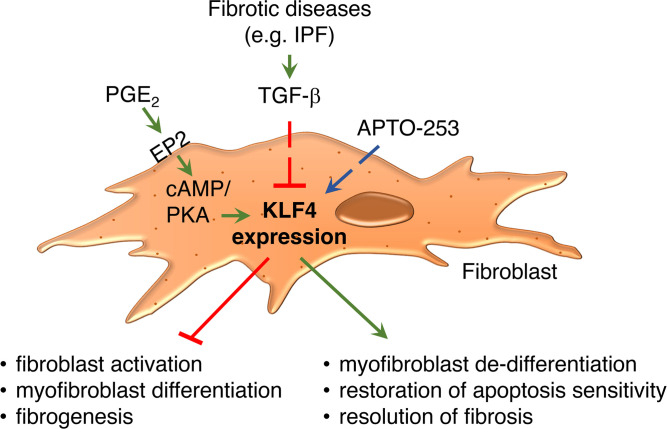

## Introduction

Fibrosis, or scarring, can develop in virtually all organs and tissues, and fibrotic diseases are estimated to account for almost half of all deaths in the United States (1). Such disorders are exemplified by idiopathic pulmonary fibrosis (IPF), the most common fibrotic lung disease, which has a poor prognosis with no cure available. Regardless of the organ site affected, fibrosis is a dysregulated response to tissue injury with shared pathophysiologic features. Key proximal elements in fibrogenesis include epithelial injury and macrophage activation. Ultimately, however, the elaboration of extracellular matrix proteins such as collagen that comprise scar tissue — the critical distal and defining element of tissue fibrosis — is mainly mediated by fibroblasts (2, 3). In response to profibrotic mediators such as TGF-β present in fibrotic disorders, fibroblasts differentiate to myofibroblasts, contractile protein-expressing mesenchymal cells that synthesize abundant quantities of collagen and resist apoptosis. Given the critical effector role of myofibroblasts, there has been substantial interest in understanding molecular pathways regulating their activation that are deregulated in fibrotic disorders and that might serve as therapeutic targets. Indeed, numerous drivers of myofibroblast activation have been shown to be overexpressed or overactive in fibrosis, but the efficacy of targeting them for inhibition is limited by their redundancy. Less is known about endogenous brakes on myofibroblast activation that are deficient in disease and which might be therapeutically augmented.

Krüppel-like factor 4 (KLF4) is a zinc finger–containing transcription factor that regulates a number of fundamental cellular processes (4, 5). It is best known for its role in controlling cell fate and differentiation (6) and for its ability to either promote or suppress growth of various types of tumor cells in a context-dependent manner (7, 8). Information about the role of KLF4 in tissue fibrosis is limited but similarly conflicting. Its expression in fibrotic lung was reported to be diminished, and it suppressed TGF-β–induced epithelial-mesenchymal transition (EMT) to myofibroblasts in a model of bleomycin-induced pulmonary fibrosis (9). By contrast, it activated Hippo signaling and promoted renal epithelial EMT as well as renal fibrosis in a model of ischemia reperfusion (10). KLF4 has been reported to both inhibit (11) and enhance (12) fibroblast differentiation to myofibroblasts in response to TGF-β. Finally, KLF4 promoted M2 polarization and TGF-β secretion in macrophages (13, 14). It thus appears that KLF4 has the potential to exert contrasting effects pertinent to fibrogenesis in distinct cell types, and its roles in fibroblasts, particularly in vivo, remain to be fully elucidated.

In the present study, we employed genetic and pharmacologic strategies to comprehensively examine the role of KLF4 in the regulation of lung fibroblast activation in vitro and in vivo. We find it to be an endogenous brake on fibroblast activation that contributes to the well-recognized antifibrotic actions of prostaglandin E_2_ (PGE_2_) and cyclic AMP (cAMP) signaling, restrains peak bleomycin-induced fibrosis, and is necessary for spontaneous resolution of fibrosis. We also administered a potentially novel small molecule inducer of KLF4 that both protected mice from peak pulmonary fibrosis and elicited resolution in a repetitive bleomycin model that is otherwise persistent. Our work thus identifies KLF4 as a pivotal brake on fibroblast activation and lung fibrogenesis whose potentiation is pharmacologically tractable, suggesting a novel therapeutic approach in fibrotic diseases such as IPF.

## Results

### KLF4 expression is diminished in fibrotic fibroblasts.

**Figure 1:**
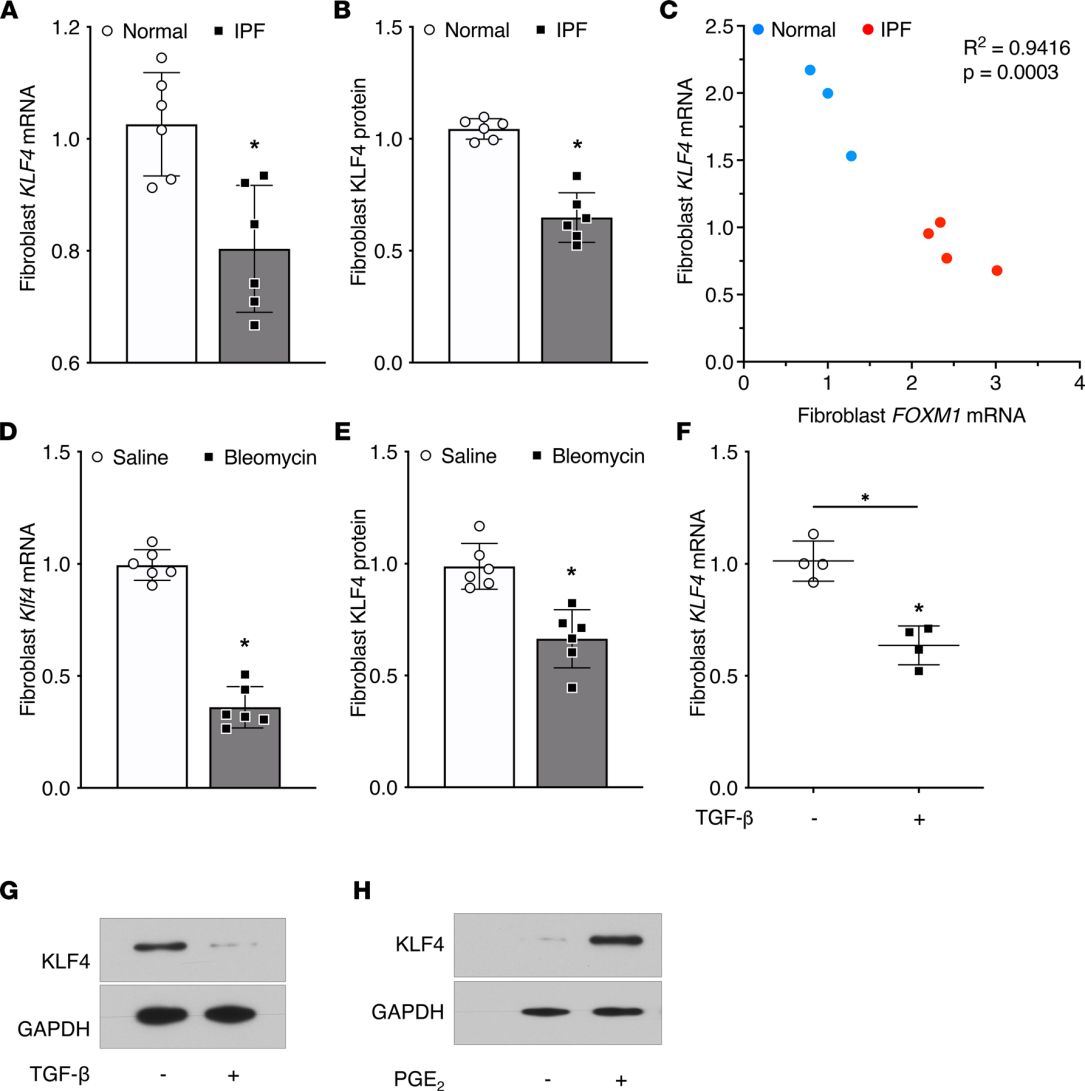
KLF4 expression is diminished in fibrotic fibroblasts. Baseline KLF4 expression in fibroblasts outgrown from lungs of patients with IPF and control nonfibrotic (normal) lungs by real-time quantitative PCR (qPCR) analysis (**A**) and protein densitometry of Western blots (**B**). (**C**) Correlation analysis between the baseline *KLF4* and *FOXM1* mRNA expression in normal (blue dots) and IPF (red dots) fibroblasts; the cell lines displayed in **C** are an unselected subset of those displayed in **A**. Baseline KLF4 expression in fibroblasts outgrown from mouse lungs on day 21 after bleomycin and saline by qPCR analysis (**D**) and protein densitometry of Western blots (**E**). KLF4 expression in CCL210 cells after 48 hours of TGF-β (2 ng/mL) stimulation analyzed by qPCR (**F**) and Western blot (**G**). Expression of KLF4 protein in CCL210 cells after 3 hours of PGE_2_ (0.5 M) treatment analyzed by Western blot (results depicted from 1 experiment representative of 3 experiments (**H**). In **A**–**C**, each symbol represents a single patient lung-derived fibroblast line, and in **D** and **E**, each symbol represents an individual murine lung-derived fibroblast line. In **A**, **B**, and **D**–**F**, mRNA and protein values are normalized to GAPDH and expressed relative to the control (normal human or saline-treated mouse). Data in **A**–**F** are shown as mean ± SEM. *P < 0.05, 2-way ANOVA.

Lin et al. reported diminished expression of KLF4 in fibrotic tissue derived from human and mouse lungs (9) and implicated KLF4 inhibition in EMT. However, the expression of KLF4 in fibroblasts from fibrotic lung tissue was not examined. We compared KLF4 expression in fibrotic and nonfibrotic (control) fibroblasts from human and mouse lung tissue (15). Fibroblasts derived from the lungs of patients with the prototypical fibrotic disease IPF exhibited significantly lower levels of KLF4 mRNA and protein (Figure 1, A and B, and Supplemental Figure 1A; supplemental material available online with this article; https://doi.org/10.1172/jci.insight.160688DS1) than did control patient-derived fibroblasts. KLF4 has been shown to oppose the transcription of the profibrotic transcription factor forkhead box M1 (FOXM1) (16), and in accordance with this recognized relationship, *KLF4* expression was inversely related to expression of *FOXM1* (15) in these fibroblast lines (Figure 1C). Next, we assessed Klf4 expression in fibroblasts outgrown from mouse lungs harvested on day 21 after bleomycin injury, a time point recognized to represent peak fibrosis in this model. Fibroblasts derived from bleomycin-treated lungs expressed significantly lower levels of Klf4 mRNA and protein than did those from control (saline-treated) lungs (Figure 1, D and E, and Supplemental Figure 1B). Mirroring what was observed in fibroblasts from fibrotic tissues, the baseline expression of KLF4 in normal human CCL-210 lung fibroblasts was significantly reduced after their differentiation into myofibroblasts by the profibrotic cytokine, TGF-β (Figure 1, F and G). On the other hand, the recognized antifibrotic mediator PGE_2_ rapidly increased KLF4 expression (Figure 1H and Supplemental Figure 2, A and B). Together, these data demonstrate that KLF4 expression is inversely associated with fibrotic activation of fibroblasts and is bidirectionally tuned by fibrogenic modulators.

### Signaling pathway by which PGE_2_ induces KLF4.

The UCSC Genome Browser indicates the presence of cAMP response element binding protein–binding (CREB-binding) sites in the human *KLF4* gene promoter (Supplemental Figure 2C). The presence of CREB-binding sites is consistent with findings from a prior study in macrophages showing a role for the cAMP/protein kinase A (PKA) pathway in *KLF4* gene induction (17). A substantial body of work from our group and others has established that the antifibrotic actions of PGE_2_ in fibroblasts are largely mediated by signaling via the Gαs-coupled PGE_2_ receptor 2 subtype (EP2)/cAMP/PKA pathway (18–20). Indeed, treatment with the EP2 agonist butaprost as well as the direct adenylyl cyclase activator forskolin increased the expression of *KLF4* mRNA and protein in a manner identical to that of PGE_2_ (Figure 1H and Supplemental Figure 2D). By contrast, peptide-mediated inhibition of PKA (myristoylated PKI_14–22_ amide) abolished the ability of PGE_2_ to increase *KLF4* mRNA (Supplemental Figure 2D). Indeed, both PGE_2_ and forskolin increased the reporter activity in fibroblasts transfected with *KLF4* promoter luciferase constructs (Supplemental Figure 2E). Together, these data implicate the EP2/cAMP/PKA pathway in PGE_2_-induced fibroblast expression of KLF4.

### KLF4 acts as a brake on fibroblast differentiation and proliferation.

**Figure 2:**
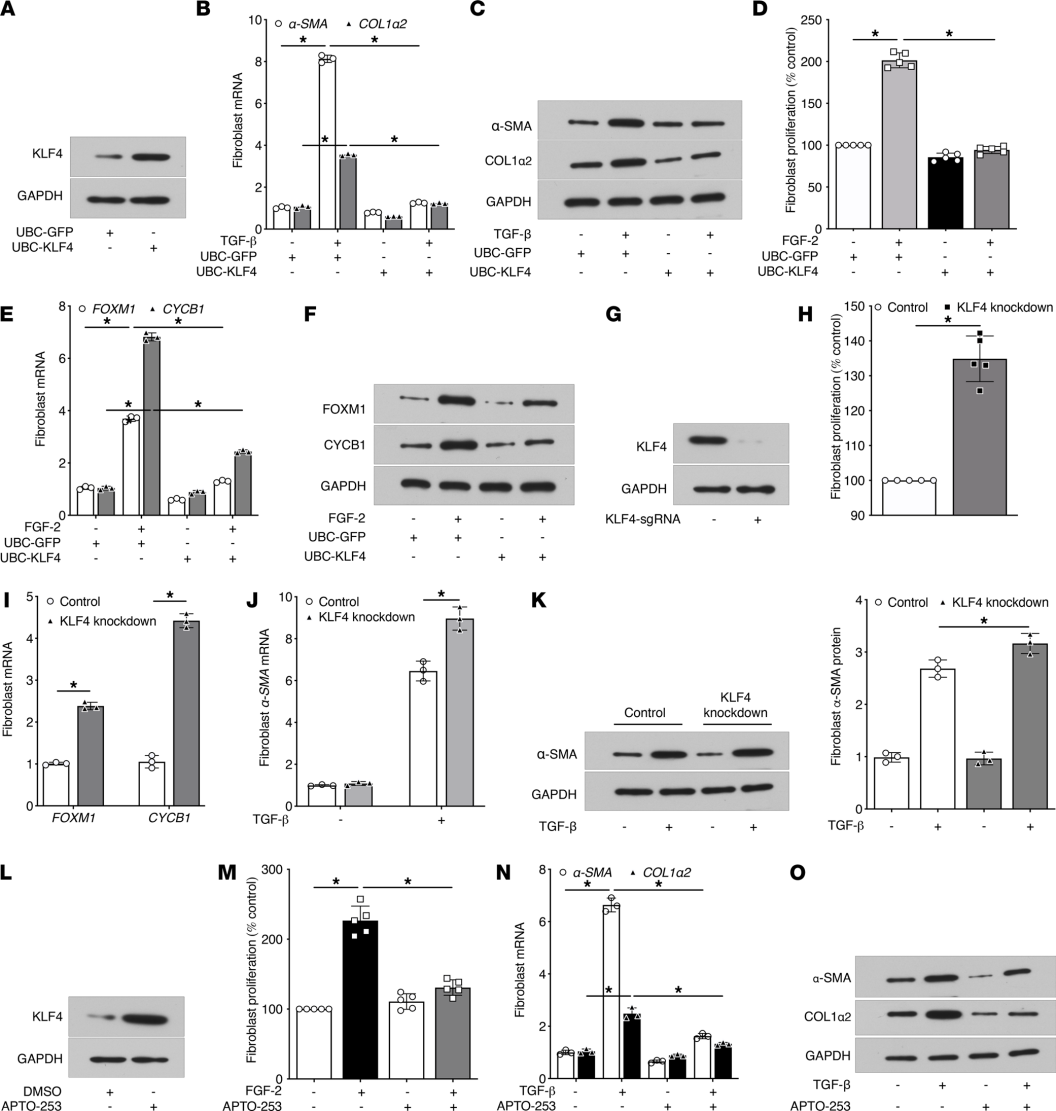
KLF4 expression regulates fibroblast differentiation and proliferation. (**A**) KLF4 protein expression in MRC5 cells 48 hours after lentiviral transduction of UBC promoter-driven GFP (UBC-GFP) or human KLF4 (UBC-KLF4). (**B** and **C**) Effect of UBC-KLF4 or -GFP on TGF-β–induced expression of α-SMA and COL1α2 mRNA at 24 hours by qPCR (**B**) and protein at 48 hours by Western blot (**C**). (**D**–**F**) Effect of UBC-KLF4 or -GFP on FGF-2–induced fibroblast proliferation as determined at 72 hours by the CyQuant NF DNA binding assay (**D**) and proliferation-associated expression of *FOXM1* and cyclin B1 (*CYCB1*) mRNA by qPCR (**E**) and protein by Western blot (**F**) at 48 hours. (**G**) CRISPR-mediated knockdown of KLF4 protein in MRC5 cells as determined by Western blot. (**H** and **I**) Effect of KLF4 knockdown on basal proliferation of fibroblasts as determined by the CyQuant NF DNA binding assay (**H**) and basal expression of *FOXM1* and *CYCB1* mRNA by qPCR (**I**). (**J** and **K**) Effect of KLF4 knockdown on TGF-β–induced expression of α-SMA analyzed at 48 hours by qPCR (**J**) and Western blot (left) and its protein densitometry (right) (**K**). (**L**) KLF4 protein induction in fibroblasts after treatment with APTO-253 (250 nM) for 36 hours. (**M**) Effect of APTO-253 on baseline and FGF-2–induced proliferation of fibroblasts as determined by the CyQuant NF DNA binding assay. (**N** and **O**) Effect of APTO-253 on TGF-β–induced expression of α-SMA and COL1α2 analyzed at 48 hours by qPCR (**N**) and Western blot (**O**). GAPDH mRNA and protein were used to normalize α-SMA, COL1α2, FOXM1, and CYCB1 expression by qPCR and Western blot, respectively. In **A**, **C**, **F**, **G**, **K**, **L** and **O**, representative Western blot of 2–3 experiments is shown. Data in **B**, **E**, **I**, **J** and **N** are expressed relative to control values and are shown as mean ± SEM from 3 independent experiments. *P < 0.05, 2-way ANOVA.

To determine the functional roles of KLF4 in fibroblast activation, we transduced normal human lung MRC5 fibroblasts with lentivirus carrying ubiquitin C (UBC) promoter–driven human *KLF4* (UBC-*KLF4*) or a control *GFP* plasmid (UBC-*GFP*). Overexpression of KLF4 protein was confirmed at 24 hours after UBC-*KLF4* lentivirus transduction (Figure 2A). Next, we determined the effect of such overexpression on the ability of TGF-β to promote fibroblast differentiation into myofibroblasts and of fibroblast growth factor-2 (FGF-2) promote fibroblast proliferation. Overexpression of *KLF4* (but not *GFP*) attenuated the capacity of TGF-β to induce expression of the myofibroblast markers *α-SMA* and *COL1* (Figure 2, B and C) and of FGF-2 to promote cellular proliferation (Figure 2D) as well as expression of proliferation-associated genes *FOXM1* and *CCNB1* (Figure 2, E and F). This was accompanied by inhibition of FGF-2–induced *FOXM1* promoter activity (Supplemental Figure 3).

To interrogate the functional consequences of diminished KLF4 expression as manifested by fibrotic fibroblasts, we employed a CRISPR/Cas9 strategy to generate a KLF4 knockdown in MRC5 human lung fibroblasts (Figure 2G). These KLF4-knockdown fibroblasts exhibited a higher degree of baseline proliferation (Figure 2H) and expression of proliferation-associated genes (Figure 2I) and of TGF-β–induced α–smooth muscle actin (α-SMA) expression (Figure 2, J and K). Together, these data show that KLF4 acts as a brake on fibroblast activation and its deficiency — as noted in fibrotic fibroblasts — favors such activation. The findings that PGE_2_ induced KLF4 and that KLF4 inhibited parameters of fibroblast activation suggested that KLF4 induction may be required for the antifibrotic actions of this prostanoid. We used KLF4-knockdown fibroblasts to evaluate this possibility. Because PGE_2_ increases KLF4 protein by 30 minutes, we pretreated both control and KLF4 knockdown fibroblasts with PGE_2_ for 30 minutes followed by addition of TGF-β. As expected, PGE_2_ pretreatment blunted the TGF-β–induced *α-SMA* mRNA expression in control fibroblasts; however, its degree of inhibition was substantially attenuated in KLF4-knockdown fibroblasts (Supplemental Figure 4). These data suggest that KLF4 contributes to the antifibrotic actions of PGE_2_.

### Treatment with APTO-253 inhibits fibroblast proliferation and differentiation.

Our data indicate that lung fibroblast KLF4 serves as a brake on fibrosis, but its expression is downregulated in fibrosis. If feasible, pharmacologic rescue of deficient KLF4 might thus be an attractive therapeutic strategy for fibrotic lung disease. The fact that KLF4 acts as a tumor suppressor that is downregulated in certain cancers (21) led to the identification of a small molecule inducer of KLF4 — APTO-253 (22) — that has undergone limited studies in both mouse models of and selected human cancers (23, 24). We therefore sought to investigate its potential therapeutic utility in lung fibrosis and began by testing its actions in fibroblasts in vitro. Pilot studies demonstrated that APTO-253 dose-dependently upregulated KLF4 expression in normal human lung fibroblasts by 36 hours, with peak effect plateauing at 500 nM (Supplemental Figure 5A). A dose of 250 nM led to a marked increase in KLF4 protein expression (Figure 2L), without any observable cytotoxicity as indicated by lactate dehydrogenase release even at 48 hours (Supplemental Figure 5B). We therefore tested the effects of this same dose of APTO-253 on fibroblast proliferation and differentiation. Pretreatment with APTO-253 reduced the capacity of FGF-2 to increase fibroblast proliferation (Figure 2M). Likewise, APTO-253 pretreatment significantly reduced TGF-β–induced expression of α-SMA and collagen type I (Figure 2, N and O). These data demonstrate that APTO-253 induces KLF4 in normal human lung fibroblasts and inhibits parameters of cellular activation.

### Conditional overexpression of KLF4 in myofibroblasts reverses their characteristic fibrotic phenotype.

**Figure 3:**
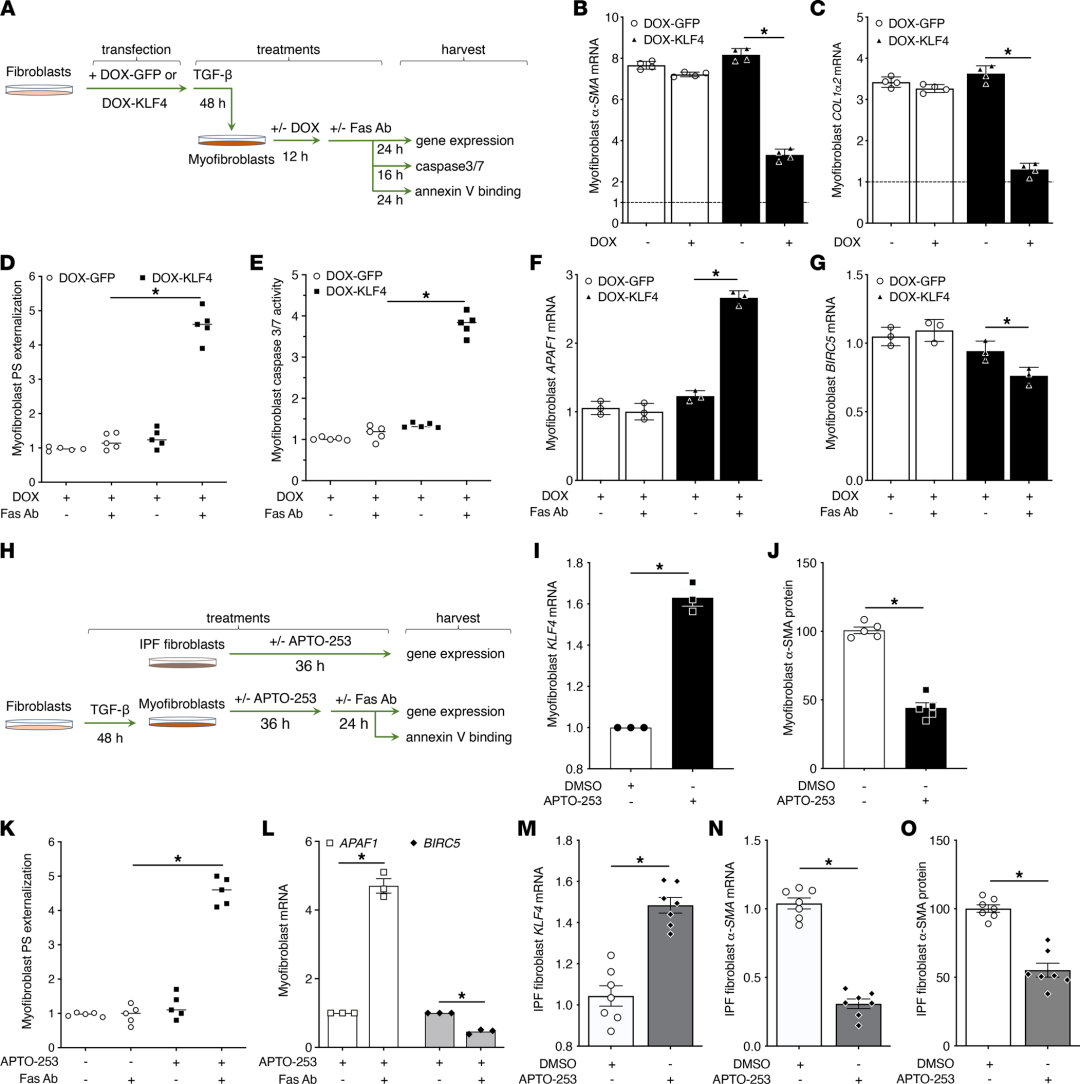
Restoration of KLF4 in elicited or IPF myofibroblasts promotes their dedifferentiation and restores apoptosis susceptibility. (**A**) Scheme illustrating the experimental layout for molecular restoration of KLF4 in myofibroblasts to evaluate their differentiation status and functional response to Fas-mediated apoptosis. (**B**–**G**) MRC5 cells stably transduced with DOX-KLF4 or DOX-GFP were treated for 48 hours with TGF-β to promote a myofibroblast phenotype. Cells were then treated with/without doxycycline (1 μg/mL) for 16 hours, and the expression of *α-SMA* (**B**) and *COL1α2* (**C**) was analyzed by qPCR at 24 hours; or cells were further stimulated with Fas Ab and assessed for relative annexin V binding at 24 hours (**D**), caspase-3/7 activity at 16 hours (**E**), and expression of *APAF1* (**F**) and *BIRC5* (**G**) measured by qPCR at 24 hours. (**H**) Scheme illustrating the experimental layout for treatment of elicited myofibroblasts or IPF fibroblasts with the pharmacologic KLF4 inducer APTO-253 and evaluation of differentiation status and functional response to Fas-mediated apoptosis. (**I**–**L**) MRC5 cells were treated for 48 hours with TGF-β to elicit myofibroblast differentiation. They were then treated with/without APTO-253 (250 nM) for 36 hours and assessed for the induction of *KLF4* by qPCR (**I**) and α-SMA protein by densitometric analysis of Western blot (**J**) or further incubated with Fas Ab and assessed for relative annexin V binding at 24 hours (**K**), with expression of *APAF1* and *BIRC5* measured by qPCR at 24 hours (**L**). (**M**–**O**) IPF fibroblasts were treated with/without APTO-253 (250 nM) for 36 hours and assessed for the induction of KLF4 by qPCR (**M**) or analyzed for the expression of α-SMA by qPCR (**N**) and protein densitometry (**O**). GAPDH mRNA and protein were used to normalize α-SMA and COL1α2 expression by qPCR and Western blot, respectively. (**M**–**O**) Each symbol represents a single patient lung-derived fibroblast line. mRNA values are expressed relative to control. (**B** and **C**) The dashed line represents the value of fibroblasts not treated with TGF-β. All data represent mean values (± SE) from 3–4 independent experiments. *P < 0.05, 2-way ANOVA.

Since patients with IPF already have established fibrosis at clinical presentation, preventing de novo differentiation — as was shown in Figure 2, B and C — is of far less translational and clinical relevance than *reversing* the differentiated phenotype of established myofibroblasts. Indeed, there is now precedent for dedifferentiation of established lung myofibroblasts (25–27), and we next wished to explore the capacity of KLF4 to do so. Treatment for 16 hours with doxycycline induced KLF4 in MRC5 normal lung fibroblasts transduced with lentivirus carrying doxycycline-inducible KLF4 (DOX-KLF4) but not in cells transduced with GFP control (DOX-GFP) (Supplemental Figure 6). As depicted in Figure 3A, we first treated these lentivirus-transduced cells with TGF-β for 48 hours to elicit differentiation into myofibroblasts. We then treated with or without doxycycline for 48 hours. Doxycycline treatment markedly reduced the expression of both *α-SMA* and *Col1α2* in DOX-KLF4 myofibroblasts but not in DOX-GFP myofibroblasts (Figure 3, B and C).

A pathogenically important hallmark of myofibroblasts believed to contribute to their accumulation in fibrotic tissue is that, unlike undifferentiated fibroblasts, they resist apoptosis (28, 29). We next asked if doxycycline induction of KLF4 in established myofibroblasts was also able to restore sensitivity to apoptosis. Fibroblasts differentiated with TGF-β and subsequently dedifferentiated with doxycycline as in Figure 3, B and C, were treated with or without a proapoptotic activating human Fas antibody (Fas Ab) for an additional 16–24 hours. The inherent resistance of myofibroblasts to apoptosis was demonstrated by the failure of DOX-GFP cells to respond to Fas Ab with any increase in annexin V binding (an indicator of phosphatidylserine [PS] externalization) (Figure 3D) or in caspase-3/7 activity (Figure 3E). By contrast, DOX-KLF4 myofibroblasts responded to Fas Ab with robust increases in both markers of apoptosis (Figure 3, D and E). Likewise, DOX-KLF4 myofibroblasts (but not DOX-GFP myofibroblasts) treated with Fas Ab exhibited an increase in expression of proapoptotic apoptotic peptidase activating factor 1 (*APAF1*) (Figure 3F) and a decrease in that of antiapoptotic baculoviral IAP repeat containing 5 (*BIRC5*) (Figure 3G). These findings demonstrate that the dedifferentiation of myofibroblasts elicited by KLF4 induction also restores their sensitivity to Fas-induced apoptosis.

### APTO-253 promotes dedifferentiation of fibrotic fibroblasts and their sensitivity to Fas-induced apoptosis.

We next sought to determine if APTO-253 could restore deficient KLF4 expression in established elicited and fibrotic myofibroblasts and concomitantly alter the fibrotic phenotype in these cells (Figure 3H). Indeed, treatment of TGF-β–elicited myofibroblasts with APTO-253 upregulated their KLF4 expression (Figure 3I) and markedly reduced their high baseline expression of α-SMA protein (Figure 3J and Supplemental Figure 7A). Moreover, it dramatically increased their susceptibility to Fas-induced apoptosis (Figure 3K) and increased expression of *APAF1* while reducing that of *BIRC5* (Figure 3L). Likewise, APTO-253 treatment of IPF fibroblasts increased *KLF4* expression (Figure 3M) and significantly reduced their high baseline expression of α-SMA mRNA and protein (Figure 3, N and O, and Supplemental Figure 7B). Taken together, these data show that APTO-253 can upregulate KLF4 expression and exert pleiotropic antifibrotic actions in fibrotic (myo)fibroblasts, as it can in normal fibroblasts.

### APTO-253–mediated suppression of fibroblast activation depends on KLF4 inducibility.

Although initially reported as an inducer of KLF4, APTO-253 has also been shown to repress expression of Myc (30), another transcription factor involved in regulating stemness and in promoting fibrosis (31). To evaluate whether KLF4 inducibility is required for the fibroblast-suppressive actions of APTO-253, we treated both control and KLF4-knockdown fibroblasts with APTO-253 followed by the addition of TGF-β. As expected, APTO-253 treatment reduced the ability of TGF-β to increase *α-SMA* mRNA expression in control fibroblasts; however, its suppressive effect was substantially attenuated in KLF4-knockdown fibroblasts (Supplemental Figure 8). Although some contribution from the modulation of alternative molecular targets such as Myc cannot be entirely excluded, these data suggest that the majority of the ability of APTO-253 to inhibit TGF-β–elicited differentiation to myofibroblasts is attributable to its induction of KLF4.

### Transgenic mice with conditional deletion of KLF4 in fibroblasts exhibit worse peak lung fibrosis.

**Figure 4:**
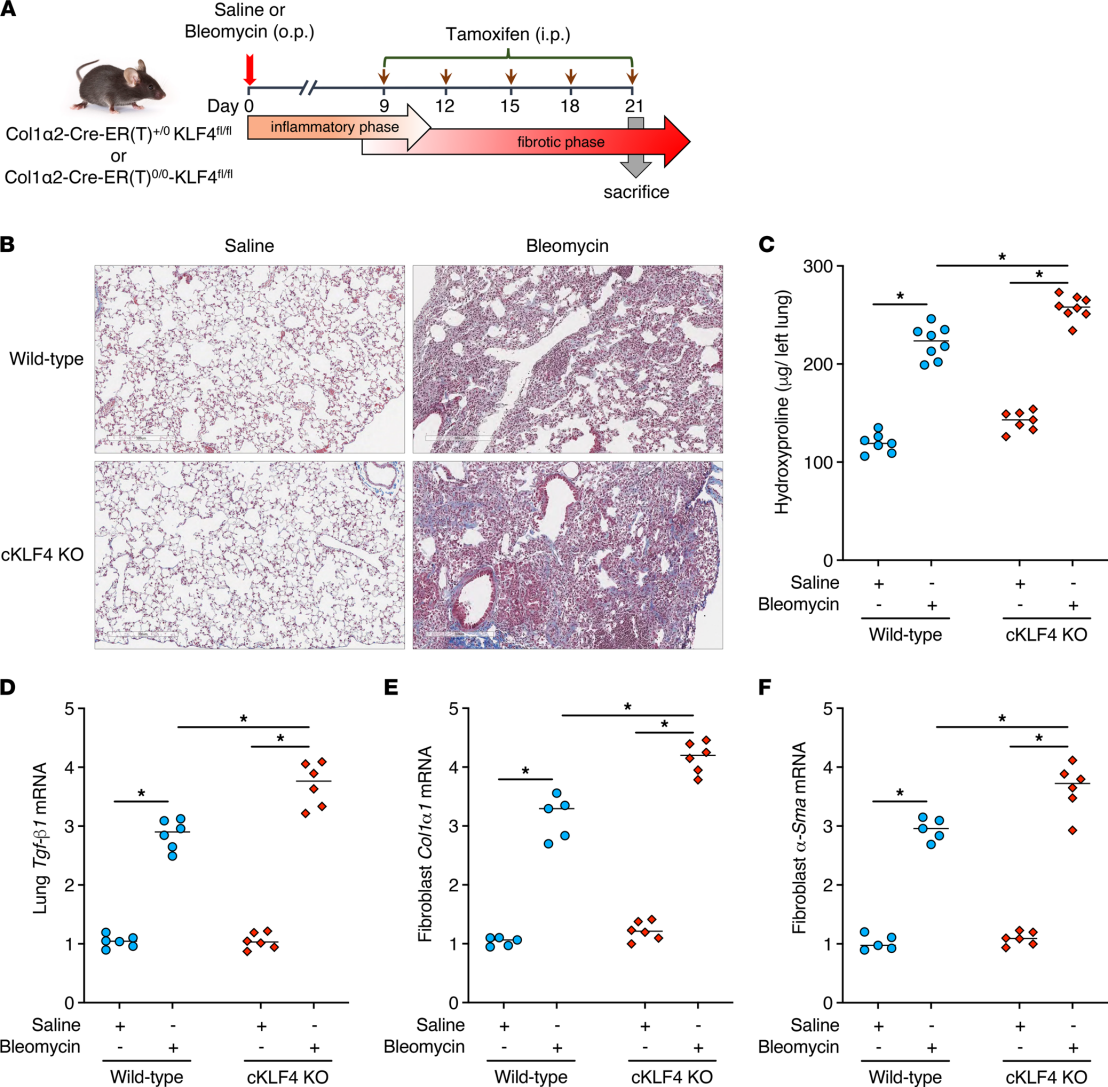
Conditional deletion of fibroblast KLF4 exacerbates peak lung fibrosis after bleomycin challenge. (**A**) Schematic illustrating the timelines for in vivo administration of bleomycin and tamoxifen and the determination of experimental endpoints at day 21. (**B**–**F**) Effect of conditional deletion of fibroblast KLF4 (cKLF4 KO) in mice treated with and without bleomycin, as reflected by Masson’s trichrome staining for collagen deposition (stains blue) (**B**), changes in the lung hydroxyproline content (**C**), and the expression of fibrotic markers *Tgf-β1* in whole lung tissue (**D**) and α*-Sma* and *Col1α1* in fibroblasts outgrown from lung tissue (**E** and **F**). Images in **B** are at original magnification of 40×; scale bar = 300 μm. In **C**–**F**, each symbol represents an individual mouse. In **D**–**F**, mRNA values are expressed relative to the WT saline control. Results are expressed as mean ± SEM, *n* = 5–8 mice per group. *P < 0.05, 2-way ANOVA with Tukey’s multiple comparisons test.

In view of our in vitro findings demonstrating that both endogenous and ectopically expressed KLF4 restrains multiple parameters of fibroblast activation, we sought to assess the importance of fibroblast KLF4 as a potential brake on lung fibrosis in vivo. To do so, we generated mice with a fibroblast-specific conditional deletion of KLF4 [Col1α2-Cre-ER(T)^+/0^-KLF4^fl/fl^ or cKLF4 KO] by crossing floxed KLF4 mice (KLF4^fl/fl^) with mice expressing Cre under a Col1a2 promoter [Col1α2-Cre-ER(T)^+/0^] and subjected them to the commonly used single-dose bleomycin-induced model of pulmonary fibrosis. This model is characterized by a postinflammatory fibrotic phase beginning at day 7 to 10 and peaking at day 21 to 28 postbleomycin. It is accepted that only experimental interventions initiated during this phase, rather than during the preceding inflammatory phase, provide information about the modulation of fibrogenesis per se. We therefore chose to administer tamoxifen every 3 days from day 9 after bleomycin administration to day 21 in order to achieve KLF4 deletion in collagen type I–expressing cells (predominantly fibro/myofibroblasts), then harvested mice for analysis at day 21 (Figure 4A). Of note, we used this same protocol previously to induce Cre-lox–mediated deletion of the *FOXM1* gene specifically in fibroblasts (15). KLF4 knockdown was verified in fibroblasts outgrown from lungs at day 21 (Supplemental Figure 9). As compared with control mice [Col1α2-Cre-ER(T)^0/0^-KLF4^fl/fl^], mice with a conditional deletion of fibroblast KLF4 during the fibrotic phase exhibited greater deposition of collagen within the interstitium, as determined both by Masson’s trichrome staining and quantitation by hydroxyproline measurement (Figure 4, B and C, and Supplemental Figure 10). Expression of *Tgf-β1* in lung homogenates and of *Col1α1* and α*-Sma* in fibroblasts outgrown from lungs at day 21 was also higher in bleomycin-treated conditional cKLF4-KO mice than in control mice (Figure 4, D–F). Together, these findings show that endogenous fibroblast KLF4 acts as a brake on pulmonary fibrogenesis in vivo as it did on cell activation in vitro.

### Fibroblast-specific cKLF4-KO mice exhibit impaired spontaneous resolution of lung fibrosis.

**Figure 5:**
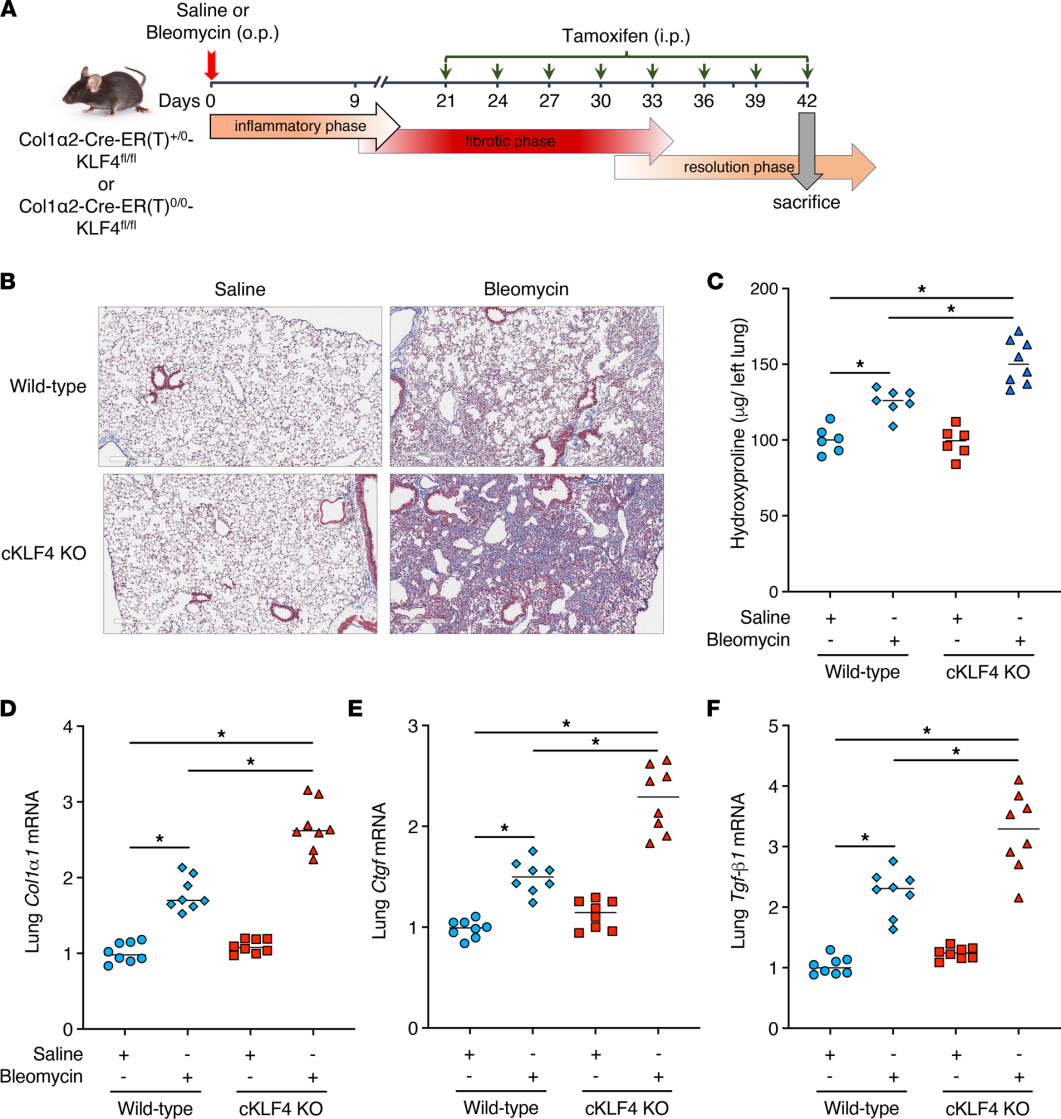
Conditional deletion of fibroblast KLF4 impairs spontaneous resolution in a transient model of lung fibrosis. (**A**) Schematic illustrating the timelines for in vivo administration of bleomycin and tamoxifen and the determination of experimental endpoints; tamoxifen administration was initiated at peak fibrosis (day 21) and continued until assessment at resolution (day 42). (**B**–**F**) Comparison of cKLF4-KO and WT mice at day 42, as reflected by Masson’s trichrome staining for collagen deposition (stains blue) (**B**), changes in the lung hydroxyproline content (**C**), and expression of fibrotic markers *Col1α1*, *Ctgf*, and *Tgf-β1* mRNA in whole lung tissue (**D**–**F**). Images in **B** are at original magnification of 40×, scale bar = 300 μm. In **C**–**F**, each symbol represents an individual mouse. Results are expressed as mean ± SEM, *n* = 6–8 mice per group. *P < 0.05, 2-way ANOVA with Tukey’s multiple comparisons test.

Although experiments such as those shown in Figure 4 are commonly terminated at 3 to 4 weeks, reflecting peak fibrosis, it is well recognized that a degree of spontaneous resolution of peak fibrosis is subsequently observed following a single intrapulmonary dose of bleomycin. Indeed, as reflected by both Masson’s trichrome staining and quantitation of hydroxyproline, our pilot studies showed substantial resolution of fibrosis by day 42 (Supplemental Figure 11). Since spontaneous resolution of fibrosis has been linked with myofibroblast dedifferentiation (25) and our in vitro studies demonstrated the capacity for ectopic expression of KLF4 to promote such dedifferentiation, we hypothesized that endogenous KLF4 within fibroblasts contributed to spontaneous resolution after single-dose bleomycin. To test this possibility, we employed the protocol outlined in Figure 5A. Briefly, we administered bleomycin at the same dose as in Figure 4A, or saline, on day 0 to cKLF4-KO [Col1α2-Cre-ER(T)^+/0^-KLF4^fl/fl^] or control [Col1α2-Cre-ER(T)^0/0^-KLF4^fl/fl^] mice and administered tamoxifen every 3 days only during the resolution phase of fibrosis, starting from day 21 postbleomycin until day 42. Mice were harvested at day 42 representing resolution. Marked spontaneous resolution was achieved by day 42 in the control mice receiving tamoxifen, as evident from morphologic restoration of alveolar architecture (Figure 5B and Supplemental Figure 12), reduced levels of lung hydroxyproline (Figure 5C), and significant declines in lung tissue expression of fibrotic genes *Col1α1*, *Ctgf*, and *Tgf-β1* (Figure 5, D–F) as compared with the values determined at peak fibrosis using this same bleomycin protocol and depicted in Figure 4. On the other hand, cKLF4-KO mice receiving tamoxifen exhibited significantly less reduction in all of these fibrotic endpoints, indicating that expression of KLF4 in fibroblasts is necessary for maximal spontaneous fibrosis resolution in this model.

### Therapeutic administration of APTO-253 protects mice from bleomycin-induced pulmonary fibrosis.

**Figure 6:**
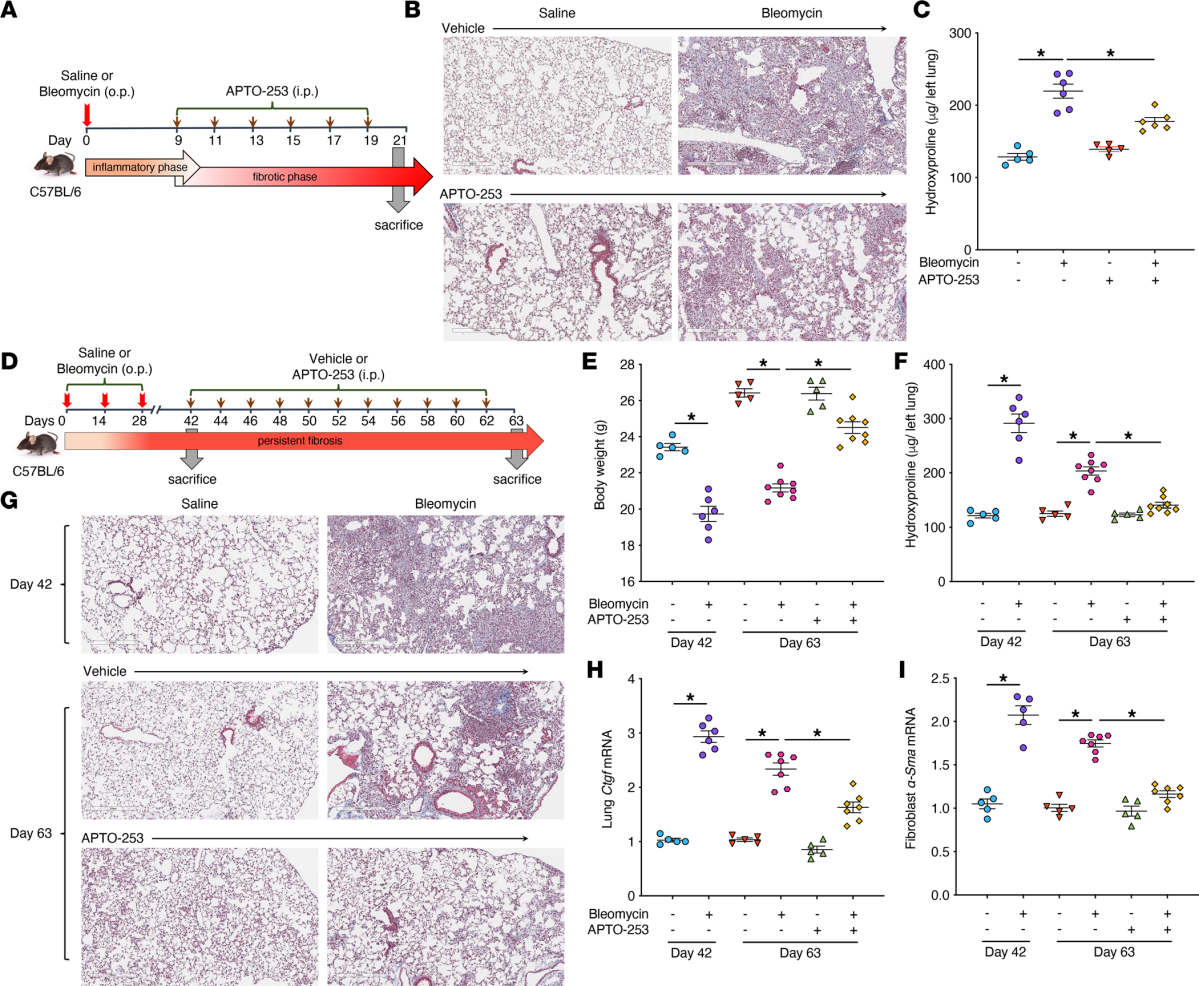
Pharmacologic restoration of KLF4 attenuates peak fibrosis and promotes resolution in a model of persistent pulmonary fibrosis. (**A**) Schematic illustrating the timelines for in vivo administration of single-dose bleomycin and APTO-253 in a transient model of lung fibrosis and the determination of experimental endpoints at peak fibrosis (day 21). (**B** and **C**) Effect of APTO-253 administration in mice treated with and without bleomycin, as reflected by Masson’s trichrome staining for collagen deposition (stains blue) (**B**) and changes in the lung hydroxyproline content (**C**). (**D**) Schematic illustrating the timelines for in vivo administration of bleomycin in a 3-dose model characterized by persistent fibrosis and administration of APTO-253 during the fibrotic phase of this model beginning at day 42 and continuing until harvest at day 63. Effect of APTO-253 administration in mice treated with and without repeated bleomycin challenge, as reflected by changes in body weight (**E**), lung hydroxyproline content (**F**), Masson’s trichrome staining for collagen deposition (stains blue) (**G**), and expression of fibrotic markers *Ctgf* in whole lung tissue (**H**) and α*-Sma* in fibroblasts outgrown from lung tissue (**I**). Images in **B** and **G** are at original magnification of 40×, scale bar = 300 μm. In **C**, **E**, **F**, and **H** and **I**, each symbol represents an individual mouse. Results are expressed as mean ± SEM, *n* = 5–8 mice per group. *P < 0.05, 2-way ANOVA with Tukey’s multiple comparisons test.

We next examined the therapeutic utility of APTO-253 administered during the fibrotic phase of the standard single-dose bleomycin mouse model of pulmonary fibrosis. APTO-253 was administered at 25 mg/kg i.p. (a dose based on that employed in a mouse model of arthritis; ref. 32) every other day beginning at day 9 postbleomycin until day 19, and lungs were harvested on day 21 (Figure 6A). As previously shown with this same model in Figure 4, bleomycin administration resulted in severe alveolar obliteration (Figure 6B and Supplemental Figure 13) and a significant increase in lung hydroxyproline content (Figure 6C). Administration of APTO-253 in control (saline-administered) mice showed no observable impact on lung architecture or hydroxyproline content. However, its administration markedly reduced bleomycin-induced fibrosis as is evident from less alveolar obliteration and significantly reduced levels of lung hydroxyproline (Figure 6, B and C). We also verified that APTO-253 administration resulted in a significant increase in lung homogenate *Klf4* expression in saline-treated as well as in bleomycin-treated mice (Supplemental Figure 14), in fact restoring the reduced level seen in bleomycin-treated mice to that of saline-treated animals. Expression of the fibrotic markers *Col1α1*, *Ctgf*, and *Tgf-β1* were also significantly reduced in the APTO-253–treated group (Supplemental Figure 15). These findings demonstrate that in vivo restoration of KLF4 restrains pulmonary fibrogenesis.

### APTO-253 shows no effect on EMT and alveolar macrophage polarization.

Fibroblasts are not the only potential cellular targets for the salutary actions of APTO-253 observed in vivo. A role for epithelial cells in pulmonary fibrogenesis is well recognized (33, 34), and KLF4 was previously reported to suppress EMT (9). We therefore evaluated the effect of APTO-253 on EMT in A549 human lung epithelial cells by treating them with this agent at 250 nM for 24 hours prior to stimulation with TGF-β for 48 hours. Consistent with an EMT response, TGF-β increased mesenchymal markers (*NCAD*, *COL4A1*, *VIM*, and *SNAIL1*) while decreasing epithelial markers (*ECAD* and *MUC1*) (Supplemental Figure 16). However, APTO-253 pretreatment had no effect on this response. Unexpectedly, *KLF4* expression in A549 cells was likewise unaffected by APTO-253 treatment (Supplemental Figure 17).

Lung macrophages are also implicated in pulmonary fibrogenesis (35, 36), and in particular M2-polarized macrophages are prime producers of the profibrotic cytokine TGF-β (37, 38). Moreover, M2 polarization in bone marrow–derived macrophages was reported to depend on KLF4 (14). We thus sought to evaluate the effects of APTO-253 on polarization of primary mouse alveolar macrophages (AMs) by pretreating them with APTO-253 (250 nM) for 24 hours followed by treatment with IL-4 for an additional 48 hours. As expected, IL-4 increased the expression of M2 markers *Arg1* and *Mrc1*, but polarization was unaffected by APTO-253 pretreatment (Supplemental Figure 18). As was observed in A549 epithelial cells, APTO-253 unexpectedly failed to induce *Klf4* in primary mouse AMs (Supplemental Figure 19). Taken together, these data demonstrate that at a dose at which it robustly induced expression of KLF4 in lung fibroblasts, APTO-253 exerted no effects on either expression of KLF4 or on relevant profibrotic functions in lung epithelial cells or macrophages in vitro.

### Administration of APTO-253 promotes resolution in a model of persistent pulmonary fibrosis.

The spontaneous resolution of fibrosis observed with the standard single-dose bleomycin protocol highlights its limitation in modeling the persistent nature of IPF. However, a 3-dose model in which intrapulmonary bleomycin is administered on days 0, 14, and 28 has been shown to yield fibrosis that is more persistent (39). Since APTO-253 protected against fibrosis in the single-dose model, we evaluated its efficacy when initiated at peak fibrosis in the 3-dose model depicted in Figure 6D. Using this approach, a maximum degree of fibrosis, which exceeded that obtained at day 21 with the single-dose model (Figure 6, A–C), was achieved by day 42, as evident from levels of hydroxyproline and Masson’s trichrome staining (Figure 6, F and G, and Supplemental Figure 20). APTO-253 (25 mg/kg i.p.) or vehicle was administered every other day from day 42 to day 60, and lungs were harvested on day 63. Consistent with a previous report (39), a substantial degree of fibrosis persisted even at day 63 in the vehicle-treated group as indicated by hydroxyproline levels and fibrotic gene expression (Figure 6, F and H). Of note, the lung hydroxyproline levels at day 63 are comparable to those at day 21 in the single-dose bleomycin model (Figure 6C). Significant loss of body weight was also apparent at day 63 in these mice (Figure 6E). By contrast, APTO-253–treated mice showed less alveolar distortion and significant reductions in hydroxyproline content and fibrotic gene expression (Figure 6, F and H). A substantial attenuation of weight loss was also noted in the treated mice (Figure 6E). Expression of α*-Sma* in fibroblasts outgrown from day 63 bleomycin lungs was markedly reduced in the APTO-253–treated group as compared with vehicle treated (Figure 6I). Collectively, these results demonstrate that KLF4 restoration via administration of APTO-253 has the potential to ameliorate pulmonary fibrosis even in a persistent model with a high degree of relevance to chronic fibrosing conditions such as IPF.

## Discussion

In view of their essential role in elaborating scar tissue, the balance of pro- and antifibrotic forces within fibroblasts and myofibroblasts is an important determinant of the progression of fibrotic diseases such as IPF. There is growing appreciation that, analogous to tumor-suppressive molecules in tumorigenesis, fibrogenesis requires that endogenous antifibrotic brakes be disabled. In this report, we have investigated the functions of KLF4 specifically within pulmonary mesenchymal cells and identify it as a pleiotropic brake on key functions of fibroblasts and myofibroblasts whose expression can be bidirectionally modulated to dictate cell activation in vitro and the outcomes of lung fibrosis in vivo.

KLF4 has previously been reported to promote murine cardiac myofibroblast differentiation via its ability to transcriptionally upregulate TGF-β1 (12). These findings are diametrically opposite of our findings in human lung cells, in which ectopic expression of KLF4 both prevented and reversed TGF-β1–induced myofibroblast differentiation. Importantly, this capacity of KLF4 to dedifferentiate myofibroblasts also resensitized them to Fas-mediated apoptosis — a phenomenon shown to be critical for their clearance from fibrotic tissues (40). These inhibitory actions on myofibroblast phenotype could be explained in part by the previous finding that KLF4 was able to bind directly to Smad3 to attenuate TGF-β1–induced differentiation of rat lung fibroblasts (11). An additional mechanism for KLF4 inhibition of differentiation likely involves its ability to negatively regulate promoter activity of the transcription factor FOXM1 (Supplemental Figure 3). Though best known for its role in promoting cell cycle genes and proliferation — a function we have verified in lung fibroblasts (15) — FOXM1 was also shown to be necessary for myofibroblast differentiation (15). A precedent for such negative regulation of FOXM1 by KLF4 can be found in gastric (41) and pancreatic (16) cancer. Ultimately, it will be of interest in future studies to explore the global impact of KLF4 on the lung fibroblast transcriptome.

Whole-lung expression of KLF4 was previously reported to be diminished in mice with bleomycin-induced fibrosis (9), a finding confirmed herein. We show here for the first time to our knowledge that KLF4 expression was significantly reduced in lung fibroblasts from both mice with bleomycin fibrosis and patients with IPF. We have also recently shown this to be the case in dermal fibroblasts from patients with systemic sclerosis (42). Indeed, its expression in IPF fibroblasts was inversely related to that of FOXM1 in IPF fibroblasts, illustrating the imbalance favoring profibrotic FOXM1 over antifibrotic KLF4 in these diseased cells. Insight into the possible mechanisms underlying downregulated KLF4 in fibrotic fibroblasts was provided by in vitro studies of the impact on KLF4 of fibroblast exposure to pertinent pro- and antifibrotic mediators. Treatment of fibroblasts with TGF-β1 downregulated KLF4 expression whereas treatment with PGE_2_ enhanced it. Since the milieu of the fibrotic lung contains increased levels of TGF-β1 (43) but decreased levels of PGE_2_(44), this imbalance of mediators could contribute to a net reduction in KLF4 expression.

Our data demonstrate that the ability of PGE_2_ to induce KLF4 expression proceeds via its ligation of the EP2 receptor with subsequent generation of cAMP and activation of PKA. Such a mechanism is not surprising since this same signaling pathway has also been implicated in the ability of this lipid mediator to inhibit lung fibroblast collagen synthesis and differentiation to myofibroblasts, to dedifferentiate myofibroblasts, and to promote myofibroblast apoptosis (45). The KLF4 promoter contains a cAMP response element (46), and a prior study likewise implicated cAMP in the ability of PGE_2_ to induce KLF4 expression within the context of M2 polarization of bone marrow–derived macrophages (46). Since the molecular mechanisms responsible for the antifibrotic actions of PGE_2_ in lung fibroblasts remain incompletely understood, we employed KLF4-deficient fibroblasts to interrogate the importance of KLF4 in mediating its inhibition of fibroblast activation. The ability of PGE_2_ to attenuate fibroblast differentiation in response to TGF-β1 was impaired in KLF4-deficient cells. Collectively, these findings suggest that KLF4 is a key molecular brake responsible, at least in part, for the antifibrotic actions of PGE_2_. Further studies with KLF4-deficient mice will be necessary to verify whether PGE_2_ relies on KLF4 for in vivo antifibrotic or proresolution activities. Of note, the KLF4 promoter also contains a peroxisome proliferator–activated receptor (PPAR) response element, and KLF4 induction has been reported in response to PPARγ agonists (47). The fact that PPARγ can inhibit fibroblast activation/differentiation and pulmonary fibrosis (48) suggests that KLF4 induction may be a conserved antifibrotic brake that mediates some of the antifibrotic actions of both cAMP-dependent and PPARγ-dependent agonists.

Consistent with the pleiotropic actions of KLF4 to oppose fibrotic pathways in vitro in fibro/myofibroblasts, we found that fibroblast-specific cKLF4-KO mice developed a degree of fibrosis following single-dose bleomycin that was worse than that of WT mice. These findings confirm that fibroblast KLF4 also acts as a brake on fibrogenesis in vivo. Since our data demonstrated that baseline expression of KLF4 was already reduced in fibroblasts isolated from mice following single-dose bleomycin (Figure 1, D and E), it is particularly interesting that even these diminished KLF4 levels appear to have been sufficient to exert some degree of restraint on fibrosis. Our results differ from a recently presented report that KLF4 deletion from fibroblasts protected from early lung fibrosis (49); however, in that study, tamoxifen was administered prior to bleomycin, and thus, those data focus on potential roles of KLF4 during the early phases postinjury, rather than focusing on the fibrotic phase of the response as we did. Such findings emphasize the context-dependent actions for KLF4.

Moreover, these fibroblast-specific KLF4-knockout mice also failed to resolve fibrosis after single bleomycin instillation, suggesting that fibroblast expression of KLF4 not only provides protection against maximal fibrotic injury but also is necessary for spontaneous resolution. Such resolution could reflect the capacity of this molecule to promote mesenchymal cell deactivation (49), apoptosis (40), or both. To our knowledge, KLF4 is the first transcription factor shown to be necessary for spontaneous resolution of fibrosis. It will be of interest in future studies to evaluate potential changes in KLF4 expression during fibrosis resolution. It is nevertheless intriguing that a recent study (50) employing transcription factor binding motif analysis found that Klf4 was the most abundant transcriptional regulator of differentially expressed genes in lung fibroblasts from young mice undergoing resolution postbleomycin but not in cells from aged mice with impaired resolution. Thus, our in vitro and in vivo findings allow us to conclude that KLF4 acts as a critical fibrotic brake.

These findings suggest the potential therapeutic benefit in fibrosis of pharmacologic agents that can restore expression of KLF4. As noted above, PGE_2_ or other cAMP agonists that increase KLF4 represent potential therapeutic candidates. However, these agents activate numerous other molecular pathways in a variety of cells, and their administration may be associated with side effects. Here we employed the small molecule APTO-253 as a means to restore KLF4. APTO-253 was originally shown to increase KLF4 in various tumors (23, 24), and its ability to do so has been attributed to Myc inhibition (30). It has been investigated in phase Ib clinical trials in myelodysplastic syndrome and acute myeloid leukemia (22), where it seems to be well tolerated. Our data demonstrate for the first time to our knowledge that APTO-253 rapidly induced KLF4 in normal lung fibroblasts as well as in TGF-β1–elicited and IPF myofibroblasts. It did so in a dose- and time-dependent manner unassociated with cytotoxicity. As was true for ectopic KLF4 overexpression, KLF4 induction by APTO-253 was associated with attenuated fibroblast differentiation and proliferation and also promoted myofibroblast dedifferentiation as well as their sensitivity to apoptotic agents. Validating KLF4 as the predominant molecular target responsible for the ability of APTO-253 to prevent myofibroblast differentiation was the finding that its action was substantially attenuated in KLF4-knockdown cells; however, we cannot exclude the possibility that effects on other molecular targets could contribute to the beneficial actions of this pharmacologic agent.

In view of its robust inhibitory actions on fibroblasts in vitro, we sought to examine the actions of APTO-253 in 2 distinct mouse models of pulmonary fibrosis. In a single-dose model of intrapulmonary bleomycin administration, administration of APTO-253 exclusively during the fibrotic phase of injury beginning at day 9 attenuated peak fibrosis observed at day 21. As this model is itself associated with spontaneous resolution of fibrosis, its fidelity as a model of chronic progressive fibrotic lung diseases such as IPF is limited. To circumvent this limitation, we also employed a repeated-dose model of intrapulmonary bleomycin in which fibrosis has been shown to be more persistent (39). Administration of APTO-253 beginning at day 42 in this model likewise attenuated the degree of fibrosis observed at day 63. In both models, the drug attenuated architectural distortion, collagen deposition, and expression of fibrotic marker genes; it also resulted in substantial reversal of the weight loss characteristic of the persistent model. Of note, APTO-253 had no evident adverse effects on the lungs of control (saline-treated) mice. Since administration of APTO-253 to WT mice in vivo would of course have the potential to affect cells other than fibroblasts, we explored its in vitro actions in epithelial cells and macrophages. Both EMT and macrophage polarization have the potential to contribute to fibrogenesis, and KLF4 has been reported to influence both processes (9, 14, 46). However, APTO-253 used at the same concentration at which it displayed efficacy in fibroblasts and myofibroblasts affected neither KLF4 expression nor the processes of EMT or M2 polarization in AMs. Together with our in vivo findings in fibroblast-specific cKLF4-KO mice, these in vitro data highlight the importance of mesenchymal cells as critical targets for the antifibrotic actions of KLF4 and for KLF4-inducing agents such as APTO-253. Additional research will be necessary to establish whether the antifibrotic actions of APTO-253 are indeed predominantly directed at fibroblasts in vivo.

In this report, we have used genetic and pharmacologic gain- and loss-of-function approaches both in vitro and in vivo to demonstrate that the transcription factor KLF4 serves as a crucial brake on lung fibrogenesis. Our data indicate that its expression within fibroblasts limits the evolution of fibrosis and contributes to spontaneous resolution of fibrosis. Pulmonary fibrosis in humans and mice is accompanied by an acquired deficiency of KLF4 within fibroblasts, consistent with the growing appreciation that fibrogenesis is associated with, and may actually require, loss of endogenous antifibrotic molecular brakes. However, its expression can be restored with the small molecule pharmacologic inducer APTO-253. Indeed, and most impressively, administration of this agent was able to reverse the persistent fibrosis seen in an experimental model felt to more closely reflect chronic fibrosing lung diseases such as IPF. Taken together, our data suggest the potential of KLF4-inducing agents as a new approach to treatment of fibrosis of the lung and perhaps of other organs.

## Methods

### Reagents.

Recombinant human TGF-β and FGF-2 were purchased from R&D Systems. Recombinant mouse IL-4 was purchased from Peprotech. Bleomycin, cell-permeable PKA inhibitor (PKI_14–22_ amide), doxycycline, human anti-Fas activating antibody (Fas Ab), and tamoxifen were purchased from MilliporeSigma. PGE_2_, forskolin, and butaprost were purchased from Cayman Chemicals. APTO-253 was purchased from MedChemExpress. Unless otherwise specified, the final concentrations of agents used for cell treatment were TGF-β, 2 ng/mL; FGF-2, 50 ng/mL; DOX, 1 μg/mL; Fas Ab, 50 ng/mL; PGE_2_, 500 nM; forskolin, 10 μM; PKA inhibitor, 10 μM; and APTO-253, 250 nM.

### Mice.

WT female C57BL/6 mice were obtained from Charles River Laboratories and used at 8 weeks of age. KLF4loxP/wt mice (B6.129S6-KLF4tm1Khk/Mmmh) were purchased from Mutant Mouse Resource & Research Centers and were intercrossed to obtain a homozygous floxed KLF4 (KLF4^fl/fl^) mouse. These KLF4^fl/fl^ mice were crossed with Col1α2-Cre-ER(T)^+/0^ mice (51) (obtained from Sem Phan, University of Michigan) as described previously (42) to generate tamoxifen-inducible fibroblast-specific cKLF4-KO mice [Col1α2-Cre-ER(T)^+/0^-KLF4^fl/fl^]. Col1α2-Cre-ER(T)^0/0^-KLF4^fl/fl^ mice were used as controls. Genotyping was performed using genomic DNA extracted from tails. Briefly, genomic DNA was extracted using REDExtract-N-Amp Tissue PCR Kit (MilliporeSigma). Cre genotyping was performed by qPCR using Cre-specific primer pair (forward 5′-CGGTTATTCAACTTGCACCA-3′ and reverse 5′-AGGTTCGCAAGAACCTGATG-3′). PCR primers designed to bind to exon 1 and exon 2 were used (5′-CTGGGCCCCCACATTAATGAG-3′ and 5′-CGCTGACAGCCATGTCAGACT-3′, respectively) to verify the homozygosity of the floxed allele for KLF4. To achieve KLF4 gene knockdown in fibroblasts (the majority of col1-positive cells), stock solution of tamoxifen (MilliporeSigma) was diluted in corn oil (MilliporeSigma) at a concentration of 10 mg/mL, and cKLF4-KO and control mice were injected i.p. with 100 μL tamoxifen solution (i.e., 1 mg per mouse).

### Bleomycin models of pulmonary fibrosis.

Single-dose administration of bleomycin results in a model in which peak fibrosis is observed at day 21, but the fibrosis is self-limiting and substantially spontaneously resolves by day 42. For experiments evaluating either peak fibrosis or spontaneous resolution, we administered a single oropharyngeal dose of bleomycin at 1.5 U/kg body weight in 40 μL saline. The control group received an equal volume of saline alone. Mice were sacrificed on day 21 or day 42, lungs were perfused, and lung lobes were harvested to assess fibrotic endpoints. Briefly, the left lung was assayed for hydroxyproline to estimate total collagen, while the right lung lobes were used to a) generate fibroblast cultures in vitro and assess their α*-Sma* expression; b) used to determine the lung expression of fibrotic markers *Col1a1*, *Ctgf* and *Tgf-β1*; and c) sectioned in order to perform Masson’s trichrome staining.

Chronic persistent fibrosis can be achieved by repeated-dose bleomycin administration, as described previously (39). For this model, bleomycin was administered at a dose of 1.0 U/kg body weight in 40 μL saline biweekly 3 times (i.e., on days 0, 14, and 28), and mice were sacrificed on both day 42 representing maximal fibrosis and day 63 representing persistent fibrosis and fibrotic endpoints assessed. In this model, body weight was also measured at these time points prior to sacrifice.

### Evaluating the role of KLF4 in models of bleomycin-induced fibrosis and resolution.

To assess the role of KLF4 in the development of peak fibrosis, tamoxifen was administered i.p. to cKLF4-KO or control mice every third day beginning at day 9 (onset of fibrotic phase) after bleomycin injury until day 21. Alternatively, 100 μL of the KLF4 inducer APTO-253, diluted in corn oil at 6.25 mg/mL, was administered i.p. to C57BL/6 mice (i.e., 25 mg/kg body weight or 0.625 mg per mouse) every other day beginning at day 9 postbleomycin until sacrifice at day 21. To assess the importance of KLF4 in the spontaneous fibrosis resolution observed at day 42 following single-dose bleomycin, tamoxifen was administered i.p. every third day to cKLF4-KO or control mice beginning at day 21 after bleomycin injury until day 42; the delay in tamoxifen initiation until day 21 is to ensure that comparable peak fibrosis had been achieved in all experimental groups. Finally, to assess the potential of therapeutic KLF4 restoration in the repeated-dose persistent model of bleomycin fibrosis, APTO-253 was administered i.p. as described above every other day from day 42 to day 63 with mice harvested at day 63.

### Lung cells and culture conditions.

Normal adult human lung fibroblasts CCL-210 (CCD-19Lu) and MRC5 (CCL171) were purchased from American Type Culture Collection (ATCC). CCL210 cells were only used up to passage 10, and MRC5 cells were used up to passage 22. For selected studies, we used fibroblasts grown as described from lung biopsy or explant specimens of patients at the University of Michigan determined to have either IPF or nonfibrotic lung under an IRB-approved protocol (15). We also obtained early-passage fibroblasts from lung tissue of saline- or bleomycin-treated mice. Briefly, lungs isolated from mice were minced with scalpels followed by digestion with 1 mg/mL collagenase A (Roche) in low-glucose DMEM (Gibco) for 1 hour at 37°C. Cell suspensions were passed through 70 μm cell strainers. Cells were washed with PBS, resuspended in complete DMEM, and seeded in culture dishes. After overnight culture, nonadherent cells were washed with PBS. Cultures were passaged for 3 to 4 times to obtain fibroblasts for experimental use. Likewise, A549 human alveolar epithelial cells (CCL-185) were purchased from ATCC. Primary AMs were obtained and purified from lung lavage fluid of C57BL/6 mice as previously described (52). Fibroblasts were cultured in low-glucose DMEM and supplemented with 10% fetal bovine serum (Hyclone) and 100 U/mL of both penicillin and streptomycin (Invitrogen), hereafter designated complete DMEM. AMs and A549 cells were cultured in complete RPMI 1640 (Gibco). All cell cultures were maintained at 37°C in a humidified atmosphere under 5% CO_2_.

### RNA isolation from cultured cells and tissue samples and qPCR.

After harvest, lung cells were lysed in TRIzol (Invitrogen). For lung tissues harvested from saline- or bleomycin-treated mice, samples were homogenized in TRIzol using the FastPrep-24 5G bead beating grinder and lysis system (MP Biomedicals). Total RNA was extracted using the RNeasy Mini Kit (QIAGEN) and quantified using a nanodrop ND-100 spectrophotometer (Nanodrop Technologies). For each sample, an equal amount of RNA was converted to cDNA using the high-capacity cDNA reverse transcription kit (Applied Biosystems). qPCR was performed using Fast SYBR green master mix (Applied Biosystems) on a StepOne Real-Time PCR system (Applied Biosystems). Expression studies for human KLF4, ACTA2 (referred to in this manuscript as *α-SMA*), *COL1α2*, *FOXM1*, *CCNB1* (referred to in this manuscript as *CYCB1*), *APAF1*, *BIRC5*, *ECAD*, *MUC1*, *NCAD*, *VIM*, and *SNAIL1*, as well as mouse *Klf4*, *Acta2* (referred to in this manuscript as *α-SMA*), *Col1α1*, *Ctgf*, *Tgf-β1*, *Mrc1*, and *Arg1*, were performed using specific primers listed in Supplemental Table 1. Relative quantification of gene expression was determined using the 2^ΔCT^ method, and human *GAPDH* and mouse β-actin were used as reference genes for human and mouse samples, respectively. mRNA levels are presented as relative to the control value, set at 1.

### Western blot.

Cultured cells were directly lysed in RIPA buffer (Cell Signaling Technology) supplemented with protease inhibitors (Roche Diagnostics) and phosphatase inhibitor cocktail (EMD Biosciences). For lung tissues, samples were resuspended in RIPA buffer and homogenized using the FastPrep-24 5G bead beating grinder and lysis system (MP Biomedicals). Samples were then centrifuged at 12,000 rpm for 15 minutes to obtain clear lysates for subsequent protein studies. Sources of antibodies were as follows: KLF4 (catalog ab129473) and α-SMA (catalog ab7817), Abcam; COL1α2 (catalog PA5-50938l), Thermo Fisher Scientific; Fas (catalog 05-201) and FOXM1 (catalog ABE1000), MilliporeSigma; and CYCB1 (catalog 4135) and GAPDH-HRP conjugate (catalog 3683), Cell Signaling Technology. All antibodies were used on both human and mouse samples at a dilution of 1:1000 except GAPDH-HRP, which was used at a dilution of 1:3000. Protein quantification was performed by densitometric analysis of blots using ImageJ software (NIH). Protein levels are presented as relative to the control value, set at 1. Full, uncut gels for all the Western blots are shown in the supplemental material.

### Plasmids.

*Homo sapiens* UBC promoter-driven KLF4 (UBC-KLF4) and GFP (UBC-GFP) constructs were generated by replacing the phosphoglycerate kinase promoter-mCherry-p2A-KLF4 cassette of pLM-mCherry-Klf4 plasmid (Addgene plasmid 23243) with the UBC promoter followed by human KLF4 coding sequence (NM_001314052.2) or EGFP (GenBank: MN832871.1) using restriction enzymes XhoI and SalI. To generate doxycycline-inducible plasmids, pLVX-TetOne-Puro Vector (Takara) was digested with EcoRI and AgeI and ligated with human KLF4 coding sequence or EGFP to generate DOX-KLF4 or DOX-GFP constructs, respectively. All plasmids were sequenced to confirm that promoters were in the correct orientation and confirm the integrity of the transgene inserts.

### Luciferase reporter assays.

The human *KLF4* (Gene ID: 9314) promoter region (–1453 to +204) was cloned into lentiviral reporter vector pEZX-LvPG04 (GeneCopoeia) to obtain pEZX-LvPG02-KLF4. This dual reporter uses Gaussia luciferase (GLuc) as the promoter reporter and secreted embryonic alkaline phosphatase (SEAP) as the internal control. To assess the KLF4 promoter activity, MRC5 fibroblasts were infected with lentiviral particles transfected with 1.5 μg of pEZX-PG04-KLF4 plasmid using FuGENE HD (Promega). Cells were cultured for 48 hours in complete DMEM prior to stimulation, and luciferase activity was determined by the Secrete-Pair Dual Luminescence Assay Kit (GeneCopoeia) according to the manufacturer’s instructions. The values obtained were presented as the relative luminescence intensity ratio of GLuc to SEAP. To generate FOXM1 promoter reporter construct, human *FOXM1* (Gene ID: 2305) promoter region (–2252 to +445) was cloned into plasmid pGL3-basic (Promega) using KpnI and XhoI restriction sites to obtain pGL3-promo_FOXM1. To assess the FOXM1 promoter activity, MRC5 cells carrying the UBC-KLF4 or UBC-GFP were cotransfected with 1.0 μg of pGL3-promo_FOXM1 and 0.05 μg of pRL-TK (a reference promoter driving Renilla luciferase) using FuGENE HD. Cells were then treated with or without 50 ng/mL FGF-2 for 24 hours. Samples were harvested and Firefly and Renilla luciferase activities were measured by the Dual-Luciferase reporter assay system (Promega) as described previously (19).

### CRISPR/Cas9-mediated knockdown.

Guide RNA targeting human KLF4 gene (sgKLF4) cloned into LentiCRISPR v2 plasmid was purchased from GenScript (target guide sequence 5′-GTGGTGGCGCCCTACAACGG-3′). This gRNA was evaluated with E-CRISP tool (http://www.e-crisp.org/E-CRISP/reannotate_crispr.html) for its specificity and efficiency. To generate KLF4-deficient MRC5 cells, cells were transduced by the lentivirus encoding Cas9 and sgKLF4 (lentiCRISPRv2-sgKLF4). After 48 hours, cells were treated with puromycin (2 μg/mL) for 3 days, and cultures were expanded and assessed for KLF4 expression by Western blotting to confirm the absence of KLF4 protein. Cells were then used for fibroblast activation studies, or aliquots were frozen in liquid nitrogen and stored at –80°C.

### Fibroblast differentiation into myofibroblasts.

Fibroblasts were seeded in 6-well plates at 5 × 10^5^ cells/well in complete DMEM, allowed to adhere overnight, and shifted to serum-free medium for 48 hours. Cells were then stimulated with TGF-β at 2 ng/mL in serum-free DMEM for 48 hours to elicit differentiation into myofibroblasts as described previously (19) and determined from the increased expression of α-SMA and COL1α2 as determined by qPCR and Western blot.

### Fibroblast proliferation.

For functional proliferation studies, fibroblasts were plated in 96-well, solid, black, flat-bottom, polystyrene plates (Corning) at a density of 5 × 10^3^ cells/well in complete DMEM; allowed to adhere overnight; and shifted to serum-free medium for 24 hours. To promote proliferation, cells were then stimulated as described previously (15) with FGF-2 at 50 ng/mL alone or in combination with APTO-253 in serum-free DMEM for 72 hours. At the time of harvest, cells were washed with PBS, and 100 μL of 1× Hank’s Balanced Salt Solution containing CyQuant NF dye (Life Technologies) was added to each well as per the manufacturer’s instructions and incubated at 37°C for 1 hour. Fluorescence intensities were measured using a fluorescence microplate reader (Tecan Infinite M1000) with excitation at 485 nm and emission at 530 nm. To assess the expression of proliferation-associated genes, assays were carried out in 6-well plates at a density of 5 × 10^5^ cells/well, then harvested at 48 hours, and expression of FOXM1 and CYCB1 was determined by qPCR and Western blot.

### Myofibroblast apoptosis.

Apoptosis in myofibroblasts was evaluated by measurement of a) annexin V binding to surface-exposed PS, b) caspase-3/7 activity, and c) expression of pro- and antiapoptotic genes. For annexin V binding studies, fibroblasts were plated in 24-well plates at a density of 5 × 10^4^ cells/well and allowed to adhere overnight. MRC5 cells carrying the doxycycline-inducible expression cassette (GFP or KLF4) were treated with TGF-β at 2 ng/mL for 48 hours for differentiation into myofibroblasts. These myofibroblasts were then treated with doxycycline for 12 hours, followed by treatment with 50 ng/mL Fas Ab for an additional 16 to 24 hours for the assessment of apoptosis. To quantify the PS exposure, samples were washed with PBS, and RealTime-Glo Annexin V Apoptosis assay reagent (Promega) was added as per the manufacturer’s instructions. Samples were then transferred into 96-well, solid, white, flat-bottom polystyrene microplates (Corning), and luminescence (which measures PS exposure during apoptosis) was recorded 1 hour later using a plate reader (Tecan Infinite 200). To detect the caspase-3 and -7 activation in myofibroblasts, cells were incubated with Caspase-Glo 3/7 substrate (Promega) followed by measurements of luminescence 1 hour later via a plate reader (Tecan Infinite 200). To complement the functional apoptosis assays, changes in transcripts encoding proapoptotic *APAF1* and antiapoptotic *BIRC5* genes were also determined in selected experiments. For studies involving APTO-253, TGF-β–generated myofibroblasts were treated with APTO-253 for 36 hours followed by Fas Ab for a subsequent 24 hours for the assessment of apoptosis as described above. For caspase 3/7 activity and annexin V binding studies, untreated or control group was set to 1.

### Statistics.

Unless specified otherwise, all data were from a minimum of 3 independent experiments. Data were reported as mean ± SEM. Group differences were compared using the unpaired 2-sided Student’s *t* test or 2-way ANOVA with post hoc Tukey’s correction for multiple comparisons, as appropriate. A *P* < 0.05 was considered statistically significant.

### Study approval.

Animal protocols were approved by the University of Michigan Committee on the Use and Care of Animals.

## Author contributions

LRP planned and performed experiments, analyzed the data, organized data for presentation, and wrote the manuscript. JMS, SKH, SMF, and JB performed experiments. MPG planned experiments, analyzed data, and wrote the manuscript.

## Supplementary Material

Supplemental data

## Version 1 07/19/2022

In-Press Preview

## Version 2 08/22/2022

Electronic publication
