# Supplementary material for: KLF4 is a therapeutically tractable brake on fibroblast activation that promotes resolution of pulmonary fibrosis
Source: JCI Insight. 2022 Aug 22;7(16):e160688. doi: 10.1172/jci.insight.160688 (PMC9462506; doi:10.1172/jci.insight.160688)
Supplement: Supplemental data [file jciinsight-7-160688-s085.pdf]

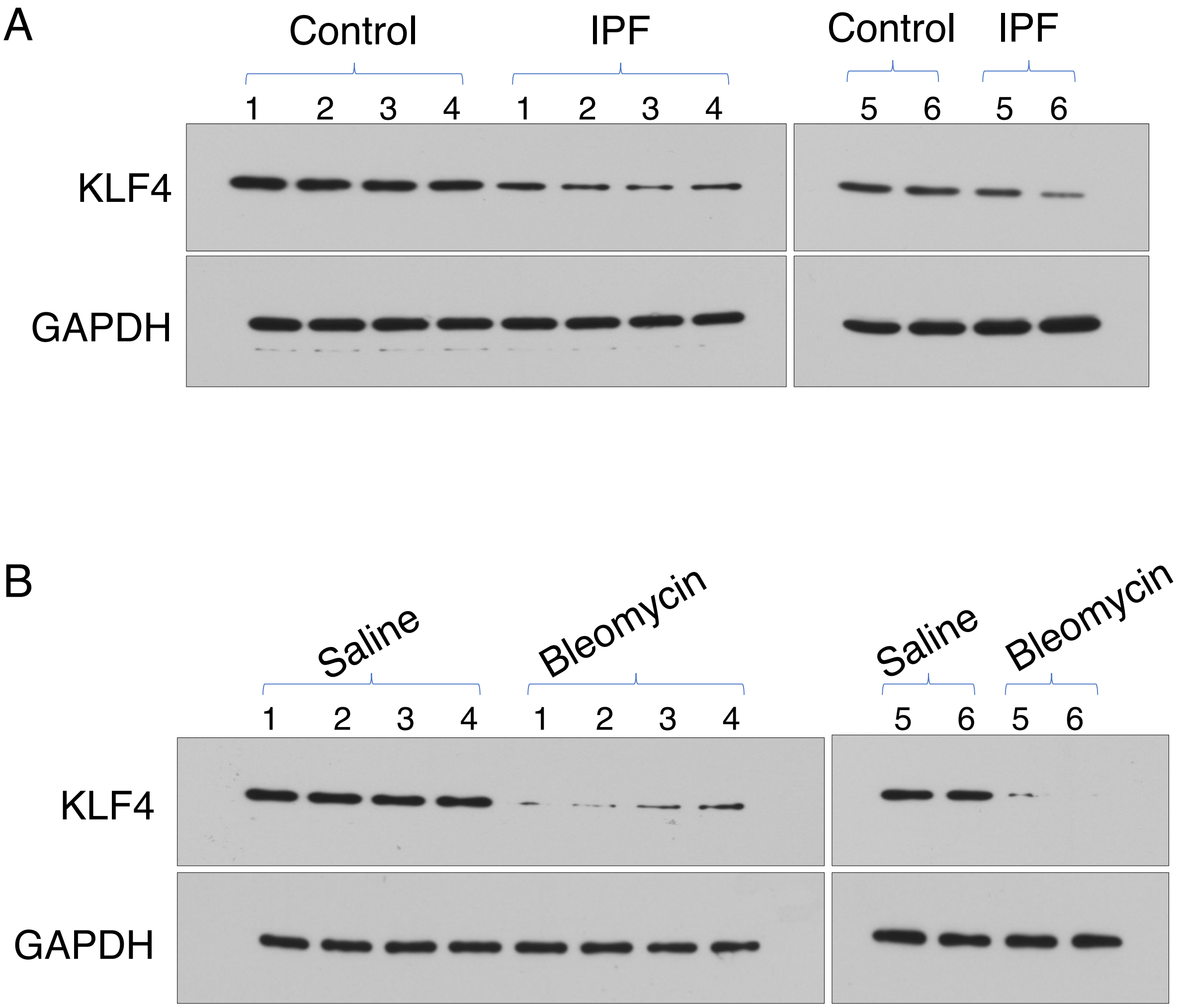

Supplemental Figure 1. Fibrotic fibroblasts display diminished KLF4 expression. (A) Baseline expression of KLF4 protein in fibroblasts grown from lung tissue derived from patients with IPF and control (nonfibrotic) lungs by Western blot. (B) Baseline expression of KLF4 protein in fibroblasts grown from lungs of bleomycin- and saline-administered mice at day 21 post-bleomycin challenge by Western blot. Each lane represents an individual patient- or murine-derived line of fibroblasts.

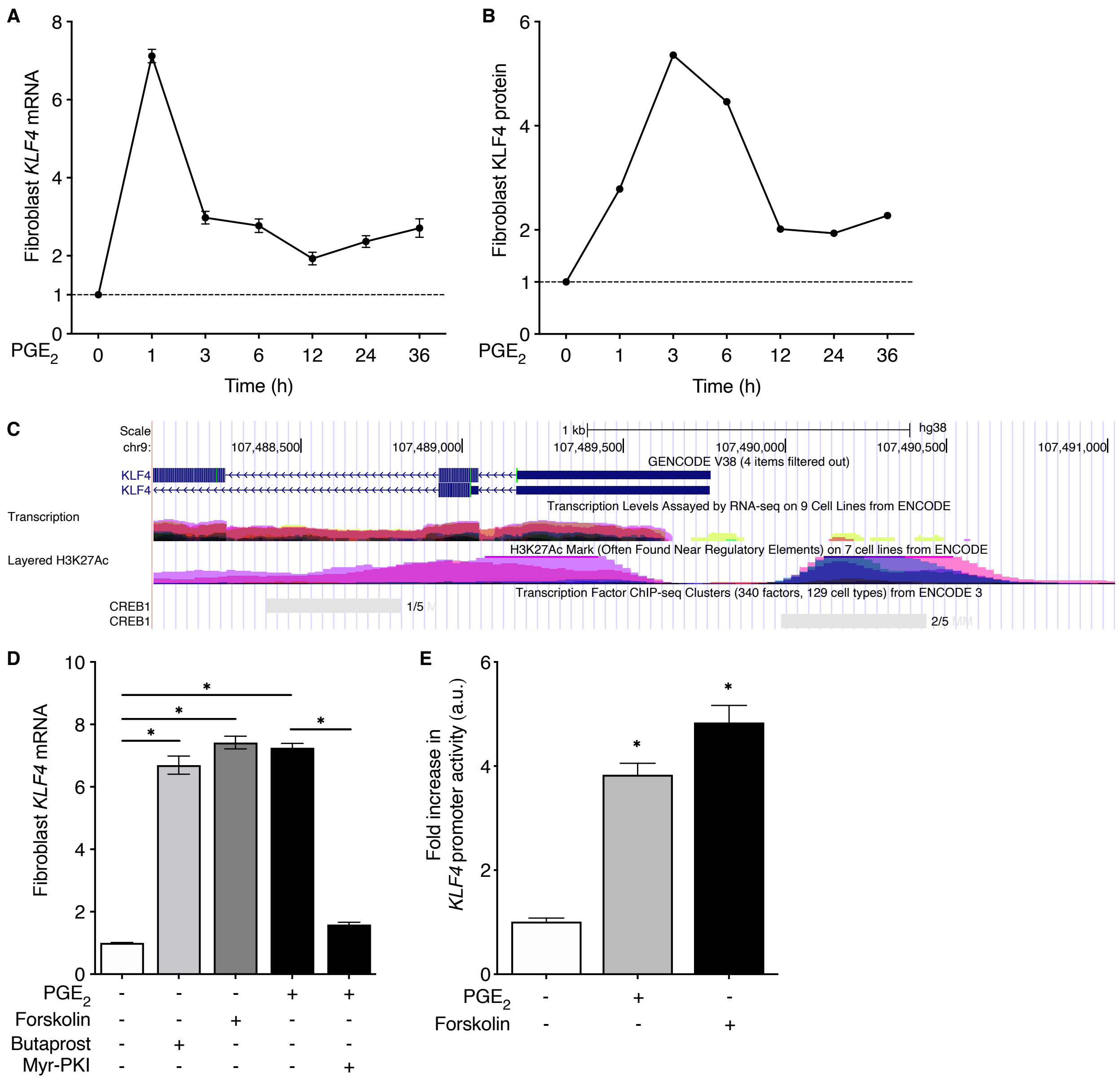

Supplemental Figure 2. Molecular pathway involved in PGE_2-induced KLF4 gene expression. (A-B) CCL210 fibroblasts were treated with PGE_2 (500 nM) and samples were harvested at regular time interval and KLF4 expression was determined by qPCR (A) and Western blot (B). (C) Depiction of CREB1 binding sites in the *KLF4* promoter region adjacent to its transcriptional start site (based on UCSC database). (D) CCL210 fibroblasts were stimulated with butaprost, forskolin, or PGE_2 (with or without $PKI_{14–22}$

amide) for 30 min. Samples were harvested and *KLF4* expression was determined by qPCR. (**E**) CCL210 fibroblasts carrying the lentiviral *KLF4* promoter were stimulated with forskolin, or PGE_2 for 1 h and KLF4 promoter activity was measured by Secrete-Pair Dual Luminescence Assay Kit. Bar graphs represent the mean ± SEM of 3 independent experiments. $^*P < 0.05$, 2-way ANOVA.

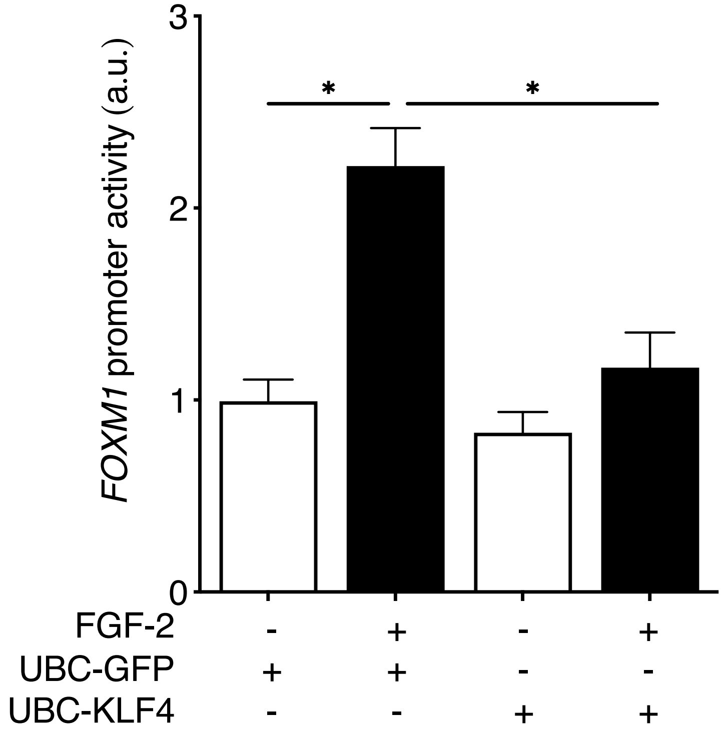

Supplemental Figure 3. Effect of forced overexpression of KLF4 on *FOXM1* promoter activity. MRC5 Fibroblasts carrying UBC-KLF4 or UBC-GFP cassette were transfected with pGL3-promo_FOXM1 for 24 h followed by FGF-2 stimulation for 48 h and *FOXM1* promoter activity was measured by the Dual-Luciferase reporter assay system. pRL-TK was used as internal control to normalize the transfection variations. Bar graphs represent the mean ± SEM of 3 independent experiments. *$P < 0.05$, 2-way ANOVA.

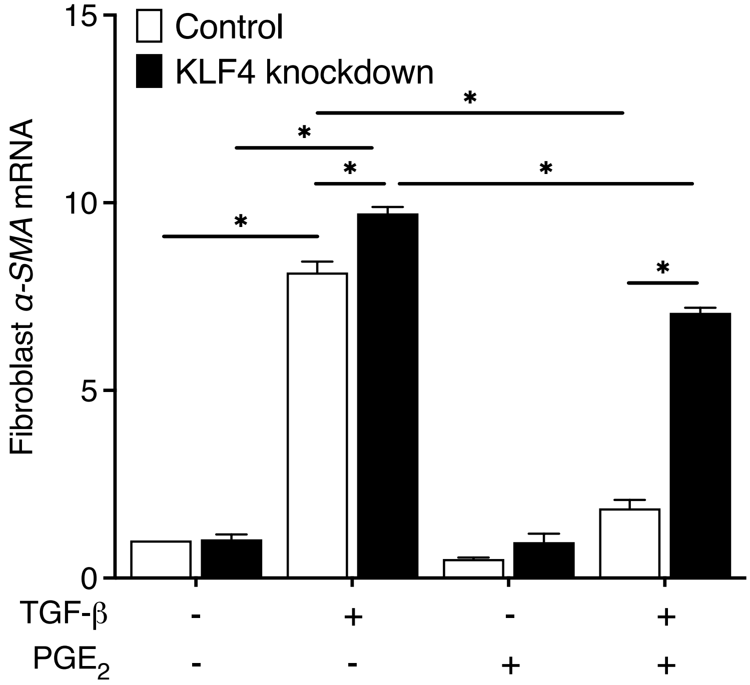

Supplemental Figure 4. Effect of KLF4 knockdown on PGE_2 anti-fibrotic actions. Control and KLF4-deficient MRC5 Fibroblasts were treated with PGE_2 for 30 min followed by TGF-β stimulation for 48 h. Expression of *α-SMA* mRNA was determined by qPCR. Bar graphs represent the mean ± SEM of 3 independent experiments. *$P < 0.05$, 2-way ANOVA.

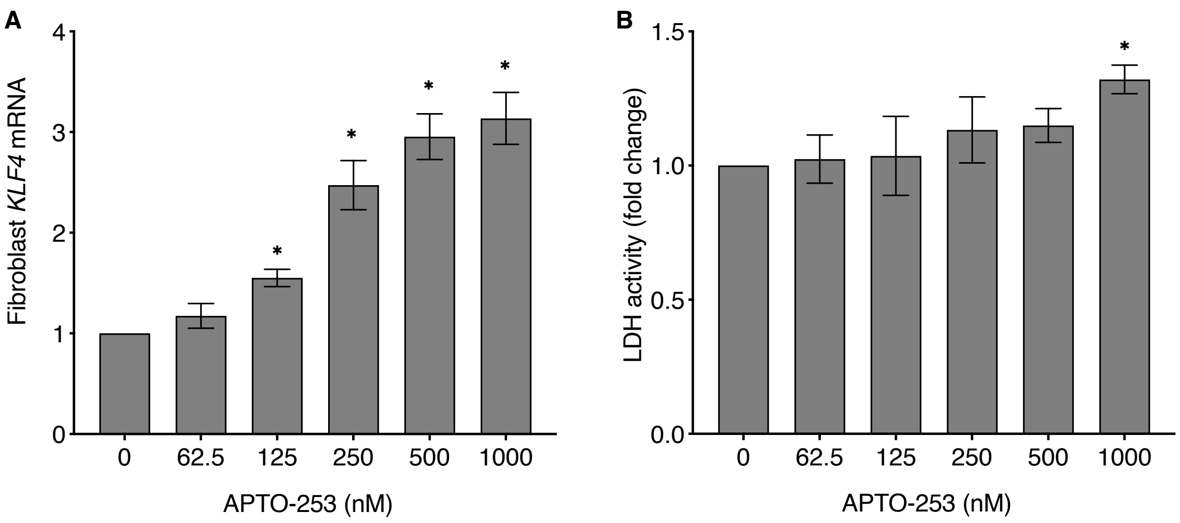

Supplemental Figure 5. Effect of APTO-253 dose on lung fibroblast KLF4 expression and viability. (A) Fibroblasts were treated with increasing concentrations of APTO-253 for 36 h and expression of *KLF4* mRNA was determined by qPCR. (B) Fibroblasts were treated with increasing concentrations of APTO-253 for 48 h, after which the medium was replaced, and cell viability was determined using the CellTiter-Glo luminescent assay kit (Promega). Bar graphs represent the mean ± SEM of 3 independent experiments. *$P < 0.05$, 2-way ANOVA.

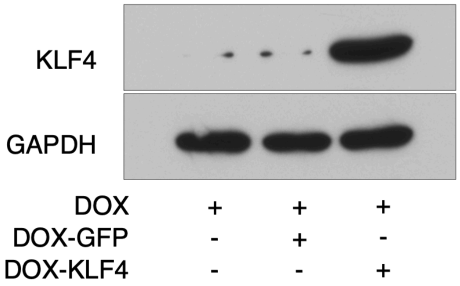

Supplemental Figure 6. DOX-mediated induction of KLF4. MRC5 normal lung fibroblasts carrying DOX-KLF4 or DOX-GFP were treated with DOX for 16 h and induction of KLF4 was evaluated by Western blot.

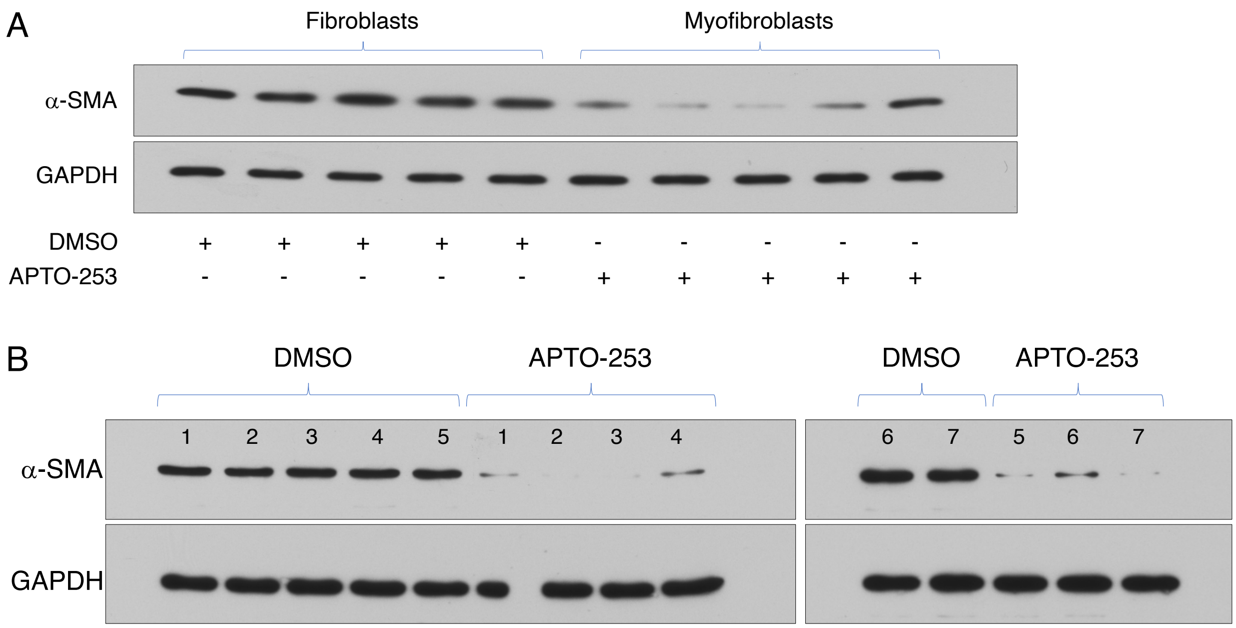

Supplemental Figure 7. Effect of APTO-253 on fibrotic fibroblast de-differentiation. (A) TGF-β-elicited myofibroblast cultures (n=5) were treated with or without APTO-253 for 36 h and expression of α-SMA was determined by Western blot. (B) IPF fibroblast lines (n=7) were treated with or without APTO-253 for 36 h and expression of α-SMA was determined by Western blot.

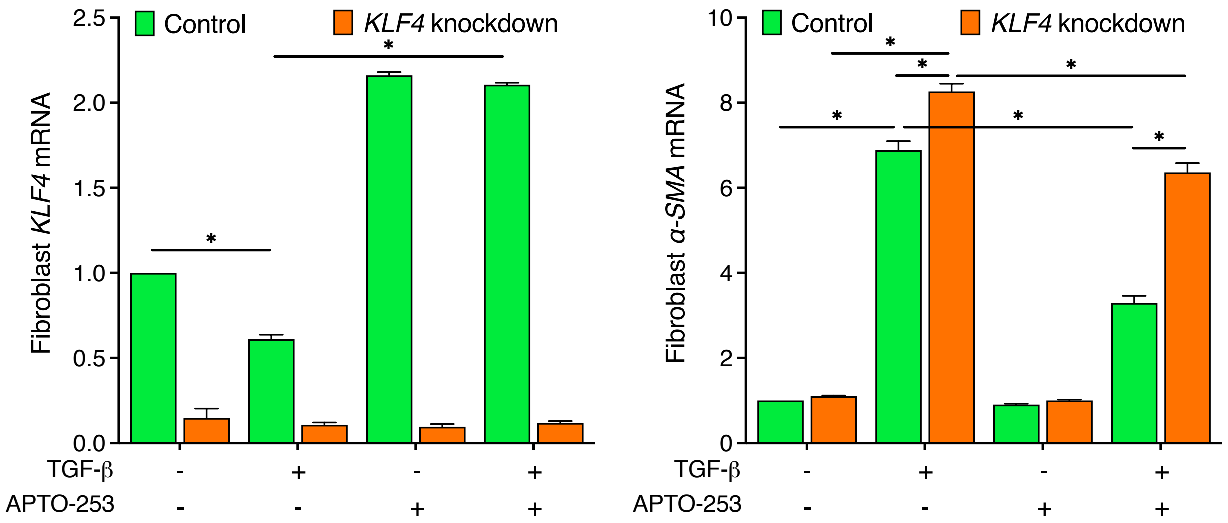

Supplemental Figure 8. Dependence on KLF4 for APTO-253-mediated suppression of fibroblast activation. (A) Control and *KLF4* (CRISPR-mediated) knockdown fibroblasts were treated with APTO-253 for 36 h followed by TGF-β stimulation for 48 h. Expression of *KLF4* (left) and *α-SMA* (right) mRNA was determined by qPCR. Although not shown in the left panel, *KLF4* knockdown was significant ($P < 0.001$). Bar graphs represent the mean ± SEM of 3 independent experiments. *$P < 0.05$, 2-way ANOVA.

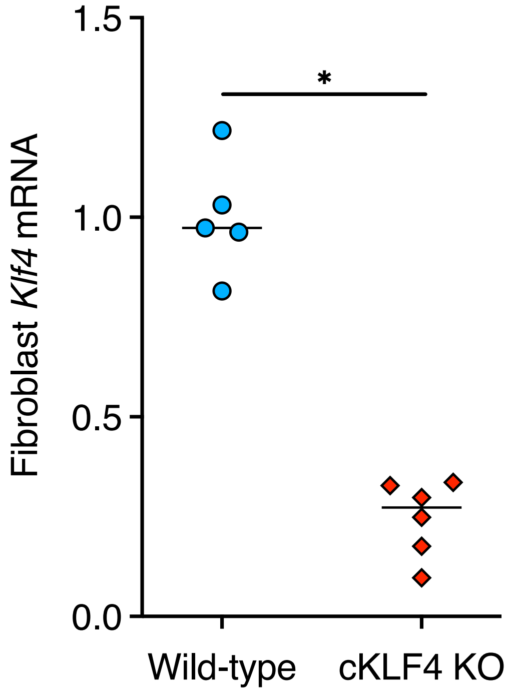

Supplemental Figure 9. Determination of KLF4 knockdown in lung fibroblasts after *in vivo* administration of tamoxifen. Both Cre^{+} $KLF4^{fl/fl}$ (Col1α2-Cre-ER(T)+/0-$KLF4^{fl/fl}$) or Cre^{-} $KLF4^{fl/fl}$ (Col1α2-Cre-ER(T)0/0-$KLF4^{fl/fl}$) mice were administered saline or bleomycin followed by tamoxifen administration every 3 days from day 9 to day 21 post-bleomycin. Fibroblasts outgrown from lungs (harvested at day 21) were assessed for *Klf4* expression by qPCR. Since *Klf4* was reduced to the same extent in cells from both saline and bleomycin treated mice, data from both groups were pooled and are represented here. Each dot represents fibroblast culture outgrown from an individual mouse lung. Data represent mean ± SEM. $*P < 0.05$, 2-way ANOVA.

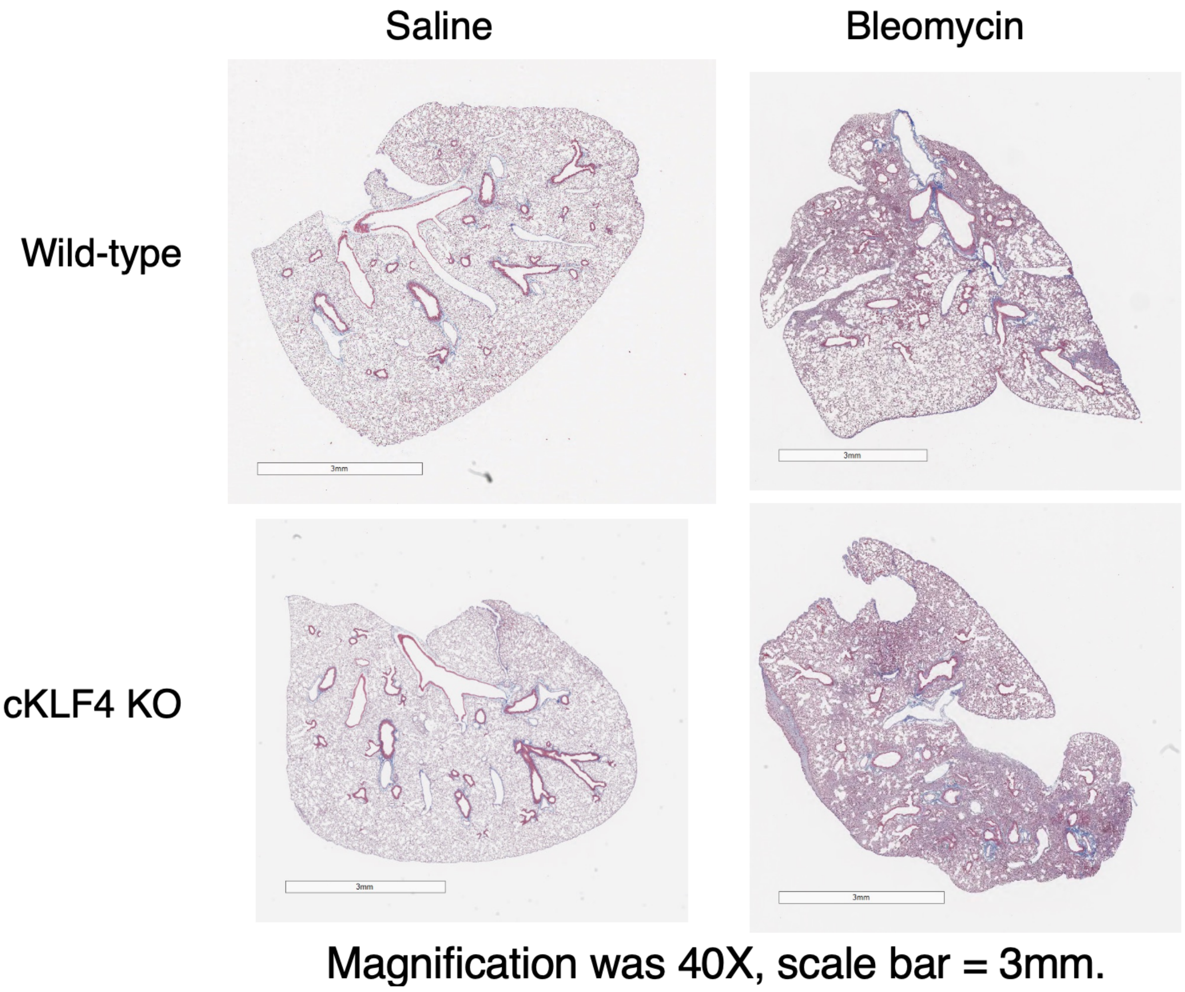

Supplemental Figure 10. Effect of conditional deletion of KLF4 in fibroblasts on lung architecture at day 21 post-bleomycin challenge. Low magnification images of Masson's trichrome-stained lung sections from mice administered saline or bleomycin followed by tamoxifen administration every 3 days from day 9 to day 21 post-bleomycin in Cre^{+} $KLF4^{fl/fl}$ (Col1α2-Cre-ER(T)+/0-$KLF4^{fl/fl}$) or Cre^{-} $KLF4^{fl/fl}$ (Col1α2-Cre-ER(T)0/0-$KLF4^{fl/fl}$) mice. Magnification: x40, Scale bars: 3 mm.

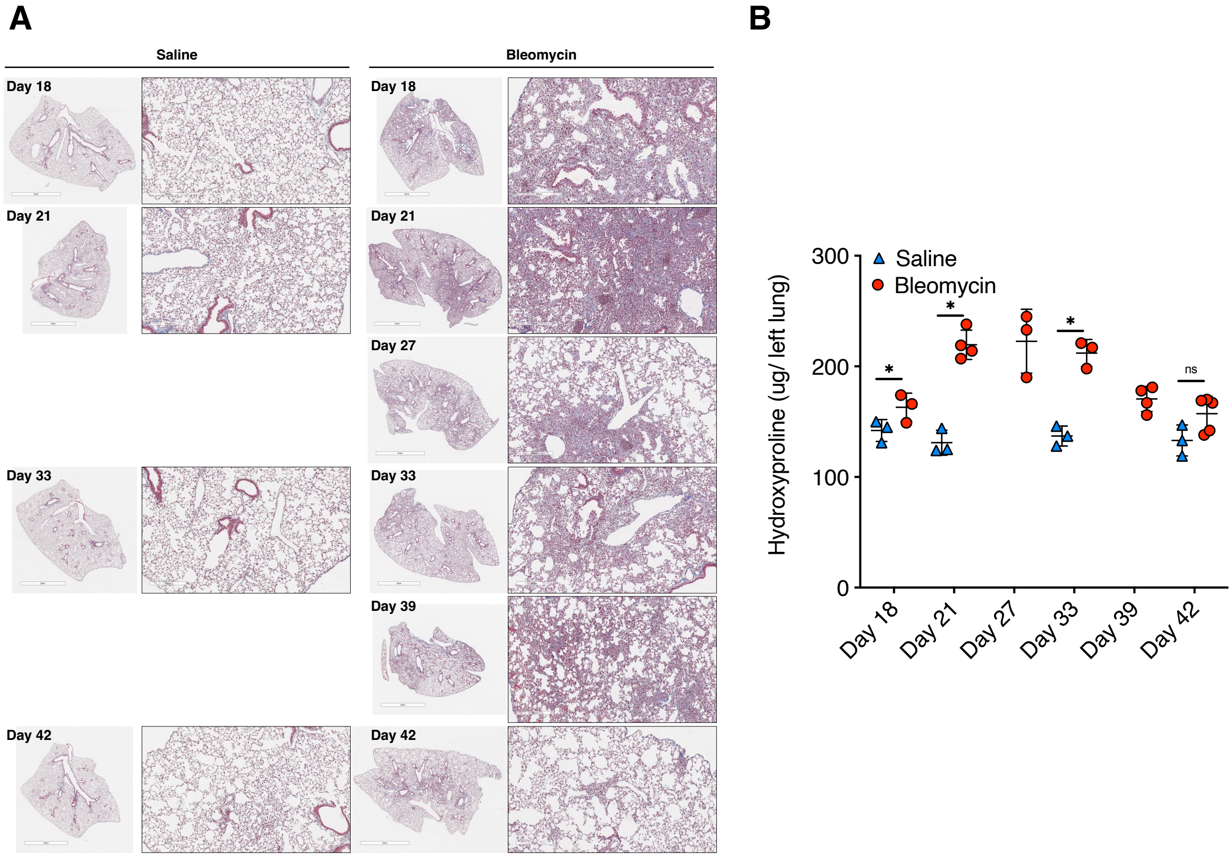

Supplemental Figure 11. Time course of bleomycin-induced lung fibrosis and spontaneous resolution. (**A**) Masson's trichrome-stained lung sections from mice administered saline or bleomycin at day 18 post-bleomycin until day 42. Magnification: x40, Scale bars: 3 mm. (**B**) Changes in the lung hydroxyproline content in mice administered saline or bleomycin at day 18 post-bleomycin until day 42.

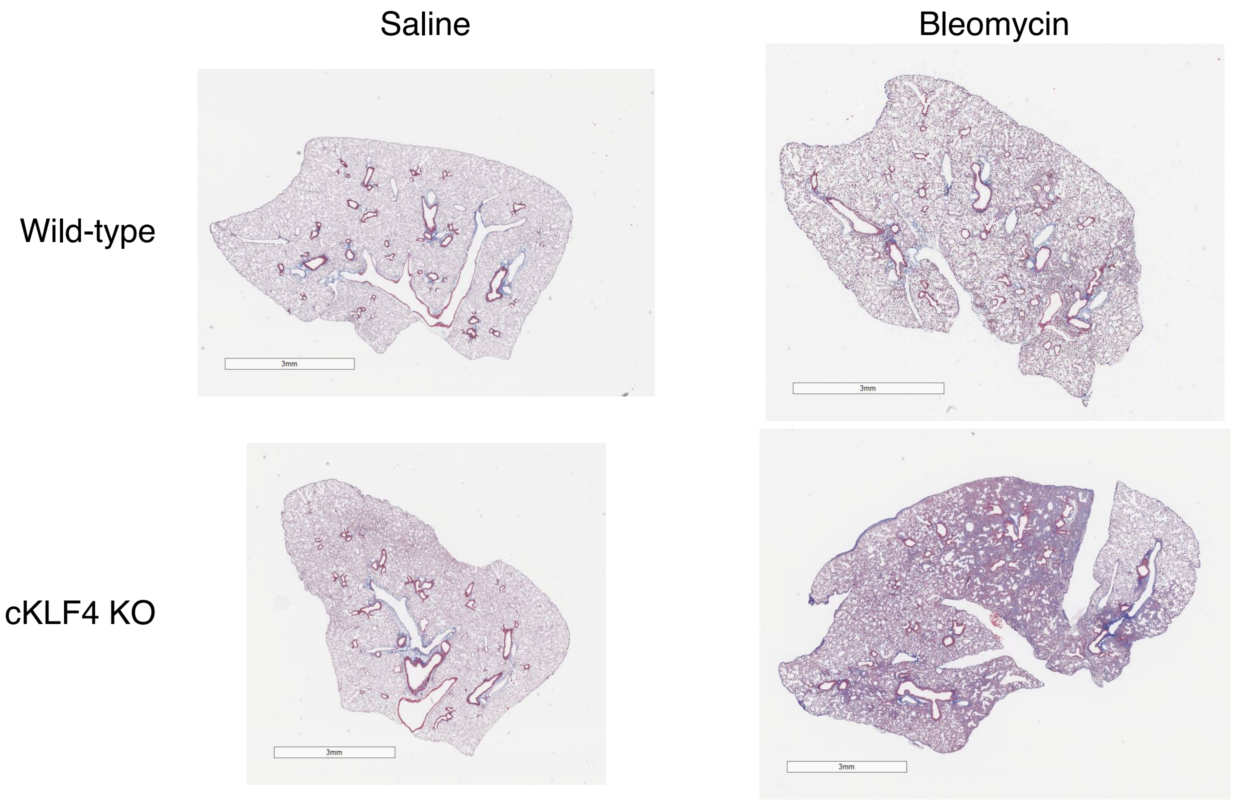

Magnification was 40X, scale bar = 3mm.

Supplemental Figure 12. Effect of conditional deletion of KLF4 in fibroblasts on lung architecture at day 42 post-bleomycin challenge. Low magnification images of Masson's trichrome-stained lung sections from mice administered saline or bleomycin followed by tamoxifen administration every 3 days from day 21 post-bleomycin until day 42 in Cre^{+} $KLF4^{fl/fl}$ (Col1α2-Cre-ER(T)+/0-$KLF4^{fl/fl}$) or Cre^{-} $KLF4^{fl/fl}$ (Col1α2-Cre-ER(T)0/0-$KLF4^{fl/fl}$) mice. Magnification: x40, Scale bars: 3 mm.

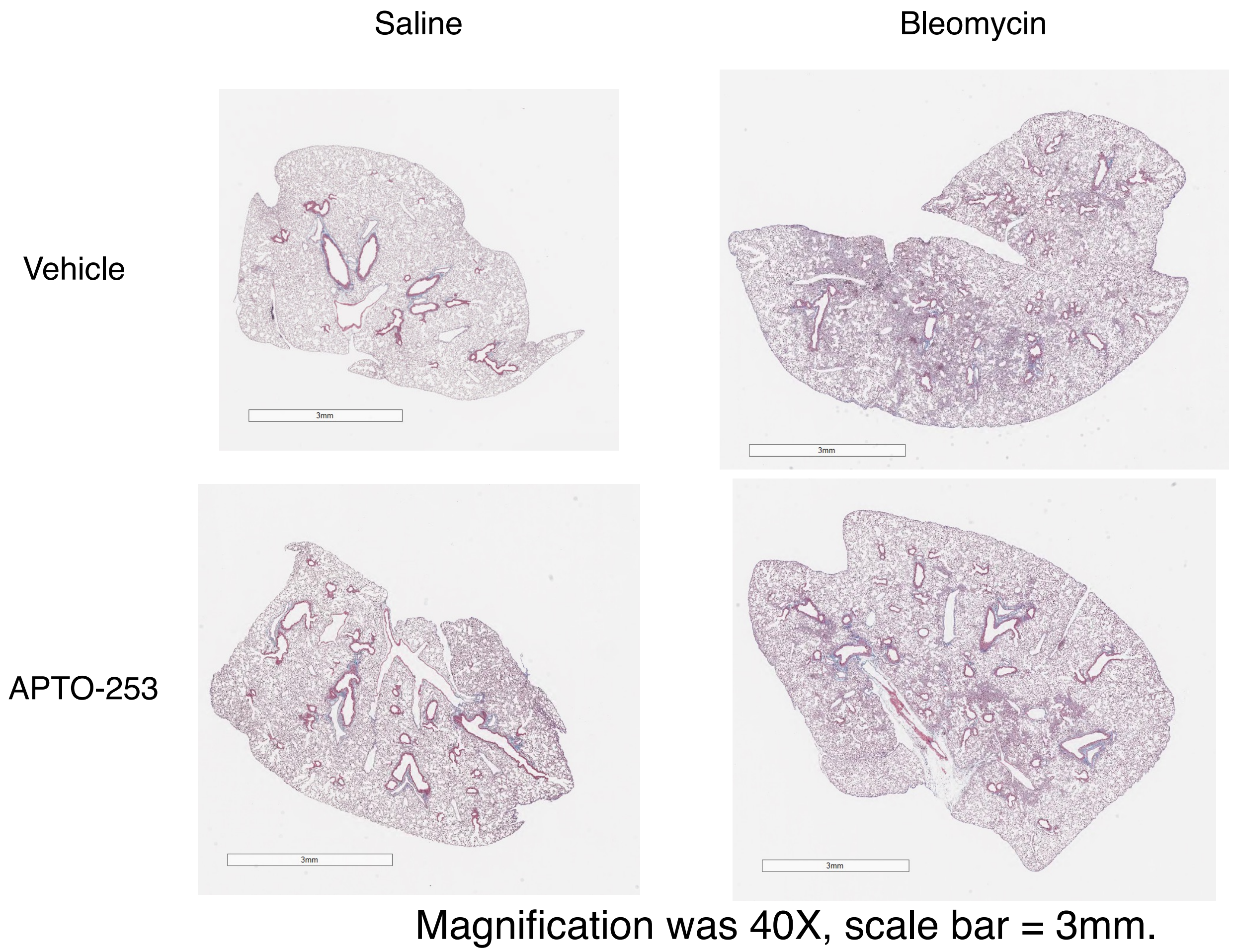

Supplemental Figure 13. Effect of APTO-253 administration on lung architecture at day 21 post-bleomycin challenge. Low magnification images of Masson's trichrome-stained lung sections from C57BL/6 mice administered saline or bleomycin followed by APTO-253 administration every other day beginning at day 9 to day 19 post-bleomycin. Magnification: x40, Scale bars: 3 mm.

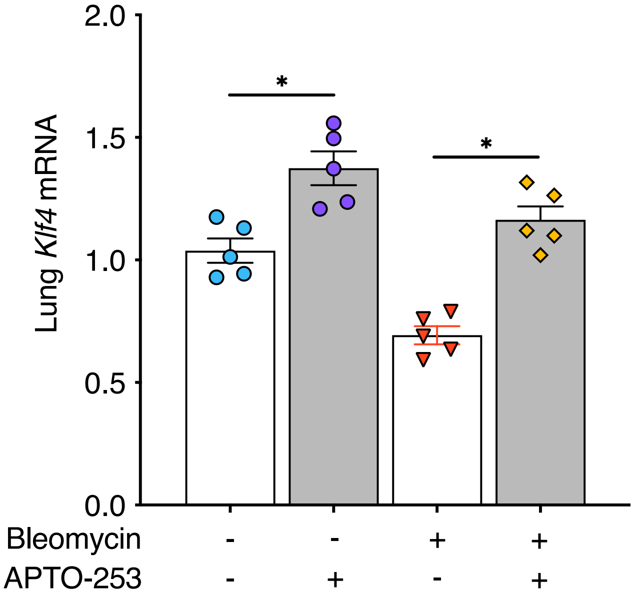

Supplemental Figure 14. Effect of APTO-253 administration on lung expression of Klf4. C57BL/6 mice were administered with saline or bleomycin followed by APTO-253 administration every other day beginning at day 9 to day 19 post-bleomycin. Lungs were harvested at day 21 and expression of *Klf4* was determined by qPCR. Bar graphs represent the mean ± SEM of 3 independent experiments. $^*P < 0.05$, 2-way ANOVA.

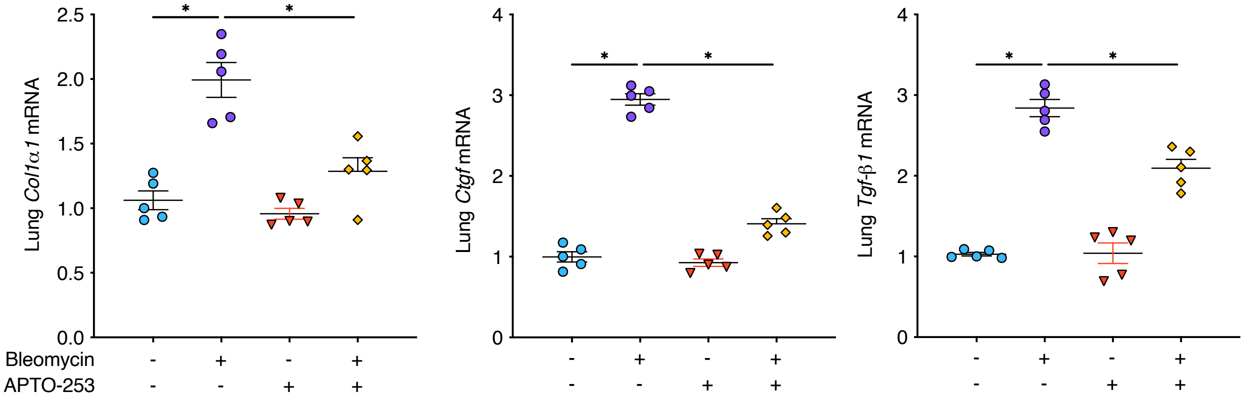

Supplemental Figure 15. Effect of APTO-253 administration on lung expression of fibrotic markers. C57BL/6 mice were treated with saline or bleomycin followed by APTO-253 administration every other day beginning at day 9 to day 19 post-bleomycin. Lungs were harvested at day 21 and expression of fibrotic markers, *Col1α1*, *Ctfg*, and *Tgf-β1* were determined by qPCR. Bar graphs represent the mean ± SEM of 3 independent experiments. *$P < 0.05$, 2-way ANOVA.

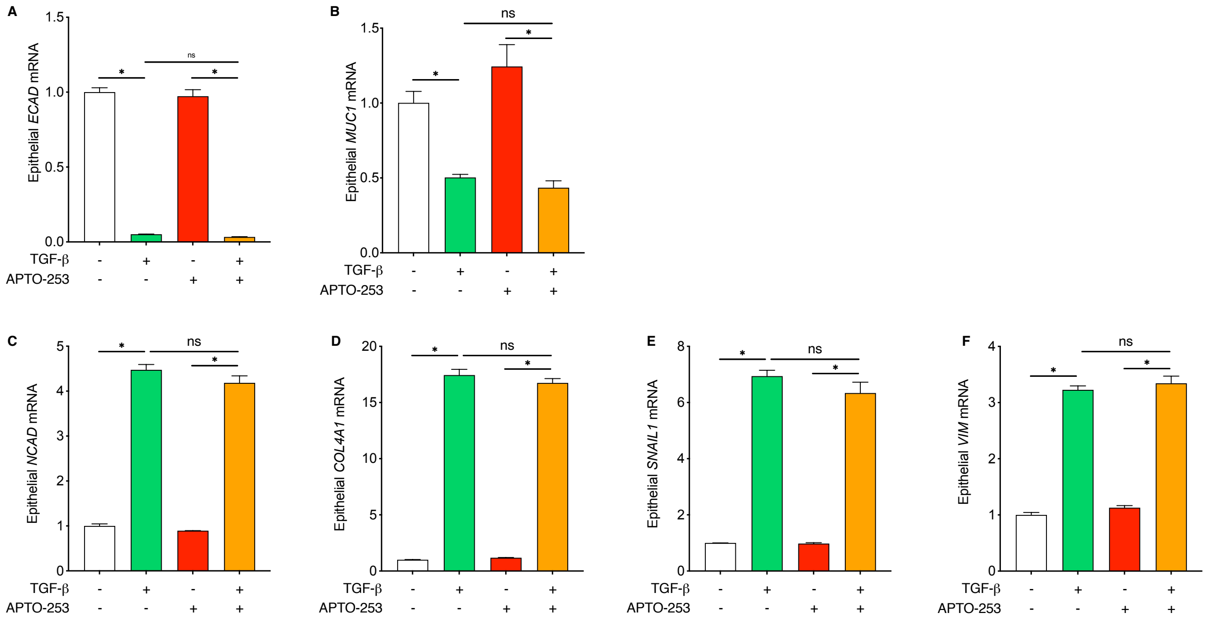

Supplemental Figure 16. Effect of APTO-253 on TGF-β-induced lung EMT. A549 lung epithelial cells were pretreated with APTO-253 for 24 h followed by TGF-β stimulation for 48 h and expression of epithelial (*ECAD* and *MUC1*) and mesenchymal markers (*NCAD*, *COL4A1*, *VIM* and *SNAIL1*) were determined by qPCR. Bar graphs represent the mean ± SEM of 3 independent experiments. $*P < 0.05$, 2-way ANOVA.

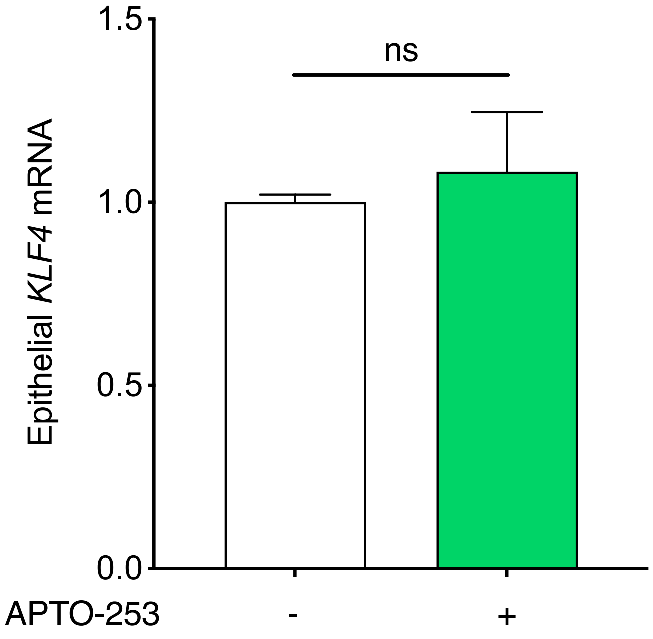

Supplemental Figure 17. Effect of APTO-253 on KLF4 induction during TGF-β-induced lung EMT. A549 lung epithelial cells were pretreated with APTO-253 for 24 h followed by TGF-β stimulation for 48 h and expression of *KLF4* was determined by qPCR. Bar graphs represent the mean ± SEM of 3 independent experiments. *$P < 0.05$, 2-way ANOVA.

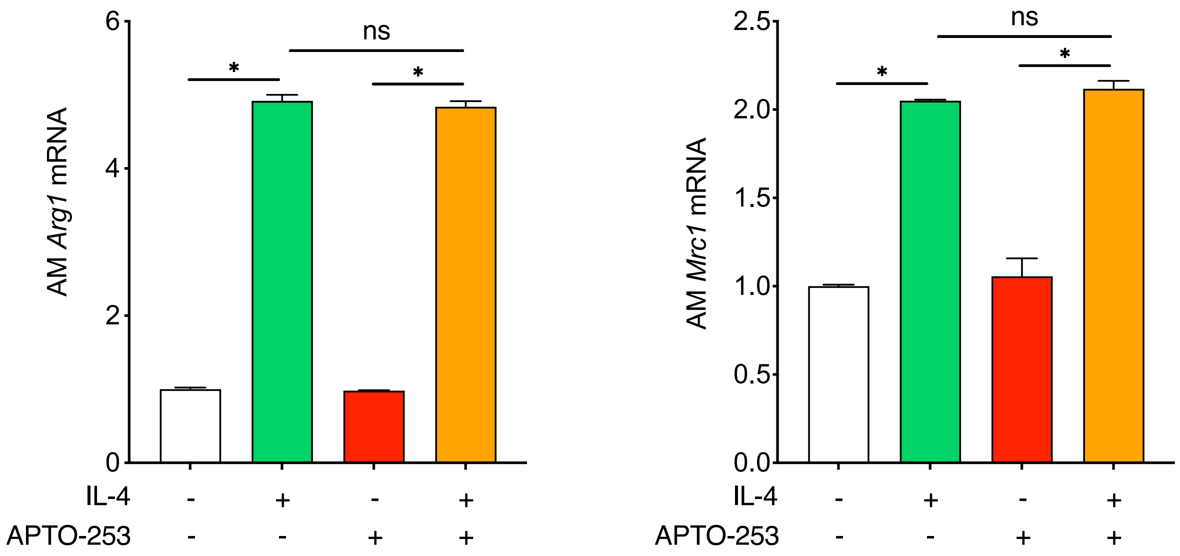

Supplemental Figure 18. Effect of APTO-253 on IL-4-induced M2 polarization of AMS. C57BL/6 mice primary AMs were pretreated with APTO-253 for 24 h followed by IL-4 stimulation for 48 h and expression of M2 markers *Arg1* and *Mrc1* were determined by qPCR. Bar graphs represent the mean ± SEM of 3 independent experiments. *$P <$ 0.05, 2-way ANOVA.

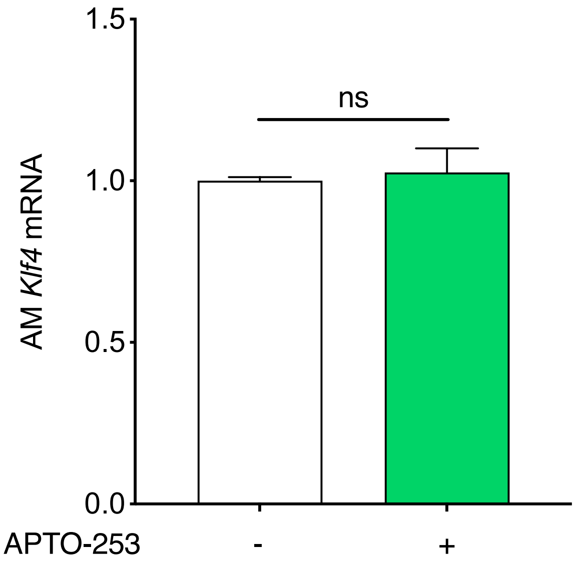

Supplemental Figure 19. Effect of APTO-253 on KLF4 induction in IL-4-induced M2 polarized AMs. C57BL/6 mice primary AMs were pretreated with APTO-253 for 24 h followed by IL-4 stimulation for 48 h and expression of *Klf4* was determined by qPCR. Bar graphs represent the mean ± SEM of 3 independent experiments. *$P < 0.05$, 2-way ANOVA.

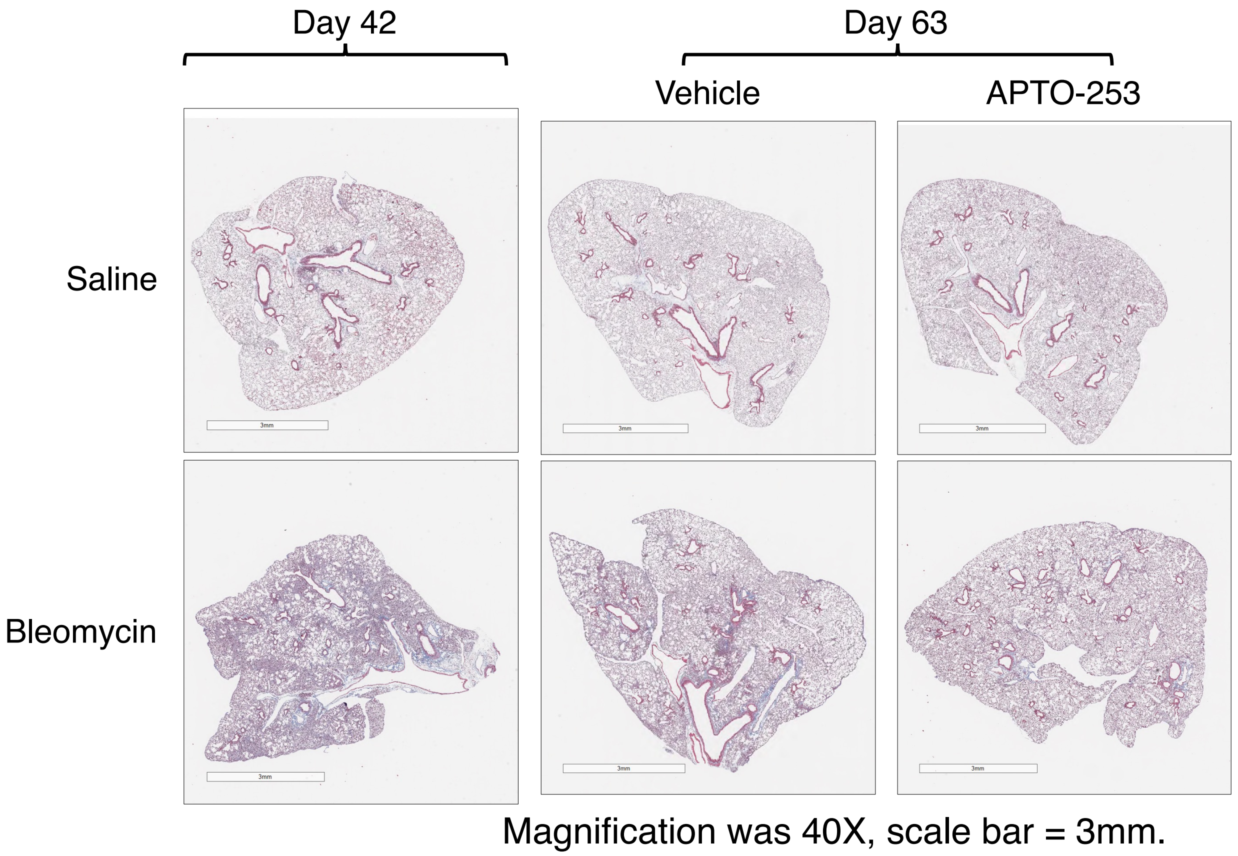

Supplemental Figure 20. Effect of APTO-253 administration on lung architecture in a model of persistent pulmonary fibrosis. Low magnification images of Masson's trichrome-stained lung sections harvested on day 63 from C57BL/6 mice administered saline or bleomycin followed by APTO-253 administration every other day beginning at day 42 to day 62 post-bleomycin. Magnification: x40, Scale bars: 3 mm.

Supplemental Table 1. Primer sequences used for qPCR

Gene	Forward primer	Reverse primer
**Human sequences**		
GAPDH	CAGCCTCAAGATCATCAGCA	ACAGTCTTCTGGGTGGCAGT
KLF4	CCAATTACCCATCCTTCCTG	ACGATCGTCTTCCCCTCTTT
α-SMA	ATCACCAACTGGGACGACAT	CATACATGGCTGGGACATTG
COL1α2	GGGTGAAATTGGAGCTGTTG	ACCAGTAAGGCCGTTTGCTC
FOXM1	GACATCTATACGTGGATTGA	GGTGAATGGTCCAGAAGGAG
CYCB1	TGTGGATGCAGAAGATGGAG	GTGACTTCCCGACCCAGTAG
BIRC5	CCACTGAGAACGAGCCAGAC	GACAGAAAGGAAAGCGCAAC
APAF1	GGAGTGCATTGGGTTTCAGT	GAGAGACCTTGGGTGTTTGC
ECAD	ACAGGATGGCTGAAGGTGAC	GAATTCGGGCTTGTTGTCAT
MUC1	CAGACGTCAGCGTGAGTGAT	GACAGCCAAGGCAATGAGAT
NCAD	ATGCCCAAGACAAAGAGACC	GTGGCCACTGTGCTTACTGA
VIM	ATGAAAGTGTGGCTGCCAAG	GTGAGGGACTGCACCTGTCT
SNAIL1	GCGAGCTGCAGGACTCTAAT	CCAGGACAGAGTCCCAGATG
**Mouse sequences**		
β-Act	GACGGCCAGGTCATCACTAT	GCACTGTGTTGGCATAGAGG
Klf4	CCAAAGAGGGGAAGAAGGTC	CGTCCCAGTCACAGTGGTAA
α-Sma	TCCCTGGAGAAGAGCTACGA	GCTGACTCCATCCCAATGAA
Col1α1	ACCTCAGGGTATTGCTGGAC	CACCACTTGATCCAGAAGGA
Ctgf	GCAGACTGGAGAAGCAGAGC	ACACTGGTGCAGCCAGAAAG
Tgf-β1	GGAGAGCCCTGGATACCAAC	ATCCACTTCCAACCCAGGTC
Mrc1	CTCTGTTCAGCTATTGGACGC	TGGCACTCCCAAACATAATTTGA
Arg1	CTCCAAGCCAAAGTCCTTAGAG	AGGAGCTGTCATTAGGGACATC
